# An *in silico* approach to identify potential downstream targets of miR-153 involved in Alzheimer’s disease

**DOI:** 10.3389/fgene.2024.1271404

**Published:** 2024-01-16

**Authors:** Sanila Amber, Saadia Zahid

**Affiliations:** Department of Healthcare Biotechnology, Neurobiology Research Laboratory, Atta-Ur-Rahman School of Applied Biosciences, National University of Sciences and Technology, Islamabad, Pakistan

**Keywords:** amyloid precursor protein, neurodegeneration, MicroRNAs, Alzheimer’s disease, miR-153

## Abstract

**Background:** In recent years, microRNAs (miRNAs) have emerged as key players in the pathophysiology of multiple diseases including Alzheimer’s disease (AD). Messenger RNA (mRNA) targeting for regulation of gene expression by miRNAs has been implicated in the annotation of disease pathophysiology as well as in the explication of their starring role in contemporary therapeutic interventions. One such miRNA is miR-153 which mediates the survival of cortical neurons and inhibits plaque formation. However, the core mRNA targets of miR-153 have not been fully illustrated.

**Objective:** The present study aimed to elucidate the potential involvement of miR-153 in AD pathogenesis and to reveal its downstream targets.

**Methods:** miRanda was used to identify AD-associated targets of miR-153. TargetScan, PicTar, miRmap, and miRDB were further used to validate these targets. STRING 12 was employed to assess the protein-protein interaction network while Gene ontology (GO) analysis was carried out to identify the molecular functions exhibited by these gene targets.

**Results:** *In silico* analysis using miRanda predicted five important AD-related targets of miR-153, including APP, SORL1, PICALM, USF1, and PSEN1. All five target genes are negatively regulated by miR-153 and are substantially involved in AD pathogenesis. A protein interaction network using STRING 12 uncovered 30 potential interacting partners for SORL1, PICALM, and USF1. GO analysis revealed that miR-153 target genes play a critical role in neuronal survival, differentiation, exon guidance, amyloid precursor protein processing, and synapse formation.

**Conclusion:** These findings unravel the potential role of miR-153 in the pathogenesis of AD and provide the basis for forthcoming experimental studies.

## 1 Introduction

Alzheimer’s disease (AD) is a neurodegenerative disorder characterized by the formation of neurofibrillary tangles (NFTs) and Amyloid-beta (Aβ) plaques in sub-cortical brain regions that eventually lead to cognitive impairment (Amber et al., 2020). Various genetic, epigenetic, and environmental factors contribute to the development of AD therefore the identification of informative biomarkers remained a significant challenge. Since the last decade, epigenetic mechanisms gained widespread prominence as the regulators of various important biological processes, and central to these processes are microribonucleic acids (miRNAs) (Filipowicz et al., 2008). miRNAs belong to the class of small non-coding RNAs that modulate gene expression post-transcriptionally either by target mRNA degradation or translational inhibition (Pu et al., 2019). miRNA:mRNA duplex formation necessitates the complementarity between eight nucleotide seed regions within both sequences. The duplex is either directed toward polyribosomes to regulate the mRNA translational process or targeted to the P-bodies for storage/degradation (Filipowicz et al., 2008). miRNAs are known to control the expression of almost 60% of protein-coding genes, therefore, these are considered important biomarkers for early diagnosis of various disorders. Their potential as potent biomarkers can be derived from unique secretory properties as they regulate the expression of multiple genes in various cell types without cell-to-cell contact (Schwarzenbach et al., 2014). Apart from their presence in tissues, miRNAs are also secreted in extracellular fluids, blood plasma, and saliva and therefore can serve as potential non-invasive markers for disease diagnosis (François et al., 2019). The preliminary evidence about the involvement of miRNAs in human diseases originated from cancer studies. Various expression profiling studies revealed the abnormal expression of different miRNAs in cancer samples as compared to the control (Calin et al., 2002).

The miRNAs that were consistently found to be deregulated in AD include; miR-9, miR-29, miR-34, miR-107, miR-181, miR-186, miR-146a, miR-155 and miR-153 (Femminella et al., 2015). The miR-153 is implicated in various diseases such as hypertension, osteosarcoma, glioblastoma, and various other cancers. miR-153 contributes toward the hypertensive state via the downregulation of KCNQ4 (Carr et al., 2016). An increase in miR-153 expression elevated neurogenesis and improved cognition (Qiao et al., 2020). Moreover, a significant reduction in the expression levels of miR-153 is also observed in early, moderate, and severe AD cases as compared to age-matched control specimens. Additionally, an inverse correlation was observed between miR-153 and Aβ plaque burden making it a potential disease biomarker and novel drug target (Long et al., 2012). Ectopic expression of miR-153-3p induced inflammation by increasing the release of IL-1β, TNF-α, and IL-6 and decreased neural stem cell differentiation via regulating GPR55 expression (Dong et al., 2023). Increased expression of miR-153 disrupted synapsin 1 in the hippocampus and impaired glutamatergic vesicle transport thus causing chronic cerebral hypoperfusion in rats (Zhang et al., 2020).

Due to the substantial role of miR-153 in neuronal disorders including AD, it is vital to identify the molecular targets associated with this very same miRNA to elucidate the underlying mechanisms leading to the disease phenotype. The data regarding the regulatory and therapeutic role of miRNAs is scarce due to the limitations of current experimental procedures (Jaberi et al., 2024). Owing to the significance of miRNAs in disease-related processes the pace of miRNA target prediction needs to be improved. Various *in silico* algorithms are available to reveal the molecular targets of a large proportion of miRNAs with relative sensitivity and specificity (Hamzeiy et al., 2014). Therefore, this study aimed to investigate the important AD-related mRNA targets of miR-153 to improve the current understanding of disease at the molecular level. AD-associated mRNA targets of miR-153 are identified via the miRanda algorithm and results are cross-validated by four other publicly available algorithms, TargetScan, PicTar, miRmap, and miRDB.

## 2 Methods

### 2.1 Targets prediction of miR-153

Web-based bioinformatic algorithm miRanda (Oliveira et al., 2017) was assessed to predict the mRNA targets of miR-153 and the mirSVR scores were assigned to each predicted target site. The sequence of miR-153 is available in the NCBI database (>LM608503.1 TPA: *Homo sapiens* microRNA hsa-mir-153precursor CTC​ACA​GCT​GCC​AGT​GTC​ATT​TTT​GTG​ATC​TGC​AGC​TAG​TAT​TCT​CAC​TCC​AGT​TGC​ATA​GTC​ACA​AAA​G TGA​TCA​TTG​GCA​GGT​GTG​GC).

The miRanda algorithm is developed for the prediction of mRNA targets and expression profiles of miRNAs available at MicroRNA.org (http://www.microrna.org); while mirSVR score is a regression model that reveals contextual features and sequence of the predicted miRNA:mRNA duplex and is directly correlated to the downregulation of miRNA and target sites of interest. *Homo sapiens* was selected as a species of choice and all the search was performed using default parameters (MFE threshold: −20 kcal/mol, scaling parameter: 4∙00, score threshold: 140.00, gap open and extend penalty: −4∙000 and −9.000 respectively).

### 2.2 Validation of results by different algorithms

The mRNA targets obtained from miRanda were further validated by four other publicly available algorithms, i.e., TargetScan, PicTar, miRDB, and MiRmap. In the TargetScan database, (Release 8, http://www.targetscan.org/), humans were selected as the species of choice. Furthermore, there were two options to find the target, i.e., by entering the gene name or miRNA name. The miRNA-153 was entered as a query and it gave two options such as miR-153–3p and miR-153–5p. Both options were explored for the target genes (Huang et al., 2020).

In the PicTar database, “PicTar target prediction in vertebrates” was selected. Following that, vertebrates was chosen as a species and then, miR-153 was selected from the dropdown menu. (http://pictar.mdc-berlin.de/) (Xue et al., 2020). In miRmap, human was selected as a species and then miR-153 was selected from the dropdown menu (https://mirmap.ezlab.org/) (Vejnar and Zdobnov, 2012).

In miRDB, humans were selected as the species of choice. Furthermore, there were two options to find the target, i.e., by entering the gene name or miRNA name. The miRNA-153 was entered as a query and it gave two selections such as miR-153–3p and miR-153–5p. Both options were explored for the target genes (http://mirdb.org/miRDB/) (Wong and Wang, 2015).

### 2.3 Protein association, functional enrichment, and post-translational modification analysis

Targets predicted by miRanda were submitted to STRING v.12 (Szklarczyk et al., 2017) (http://string-db.org/) database to explore the functional association networks of target proteins using UniProt accession numbers. *Homo sapiens* was selected from the given list of species. Biological processes, cellular localization, molecular functions, and miRNA targets of the specific miR-153 affected proteins were investigated by GO analysis and microRNA target analysis using the WEB-based Gene SeTAnaLysis Toolkit (WEBGESTALT) (Wang et al., 2017). Swiss-Prot accession numbers of miR-153 target proteins were employed for enrichment analysis.

The phosphorylation modification sites were predicted for the identified target proteins, using NetPhos 3.1 server (www.cbs.dtu.dk/services/NetPhos-3.1) (Arshad et al., 2018) while S-nitrosylation, and N and O glycosylation sites were predicted using GPS-SNO http://sno.biocuckoo.org (Mazina et al., 2023), NetNGlyc 1.0 www.cbs.dtu.dk/services/NetNGlyc/(Azevedo et al., 2018), and NetOGlyc 4.0 www.cbs.dtu.dk/services/NetOGlyc/(Kwan et al., 2021), respectively. Default settings were used for the analysis of posttranslational modification (PTM) sites and the predictions having output scores above 0.5 were only selected to avoid the possibility of false positive results. The FASTA sequence of the targeted proteins was acquired from the NCBI protein database (https://www.ncbi.nlm.nih.gov/pubmed/).

## 3 Results

### 3.1 Targets prediction of miR-153

miRanda algorithm returned 5,810 targets for miR-153 which were further screened to identify the targets involved in AD pathophysiology using a literature search. Five of the 5,810 targets found to be most relevant with AD include; sortilin-related receptor 1 (SORL1), amyloid precursor protein (APP), phosphatidylinositol binding clathrin assembly protein (PICALM), upstream stimulatory factor 1 (USF1) and presenilin-1 (PSEN1). miSVR scores indicated that miR-153 downregulates all the target genes. The results were cross-validated by four different freely accessible software TargetScan, PicTar, miRmap, and miRDB. It is observed that all five targets were not predicted by all the software (Table 1). APP and PSEN1 are already reported to be affected by miR-153 so we used SORL1, PICALM, and USF1 for further analysis.

**TABLE 1:** miR-153 targets and their miSVR scores predicted by miRanda and validated by different software.

Sr No.	miR-153 targets	miSVR score	Algorithms
1	APP	−1.2559	miRanda, Target Scan, PicTar, miRmap
2	SORL1	−0.6425	miRanda, Target Scan, MIRDB, miRmap
3	PICALM	−0.1180	miRanda, Target Scan, miRmap
4	USF1	−0.2466	miRanda, PITA, miRmap
5	PSEN1	−0.1895	miRanda, miRmap

### 3.2 Protein association network and functional analysis

**FIGURE 1:**
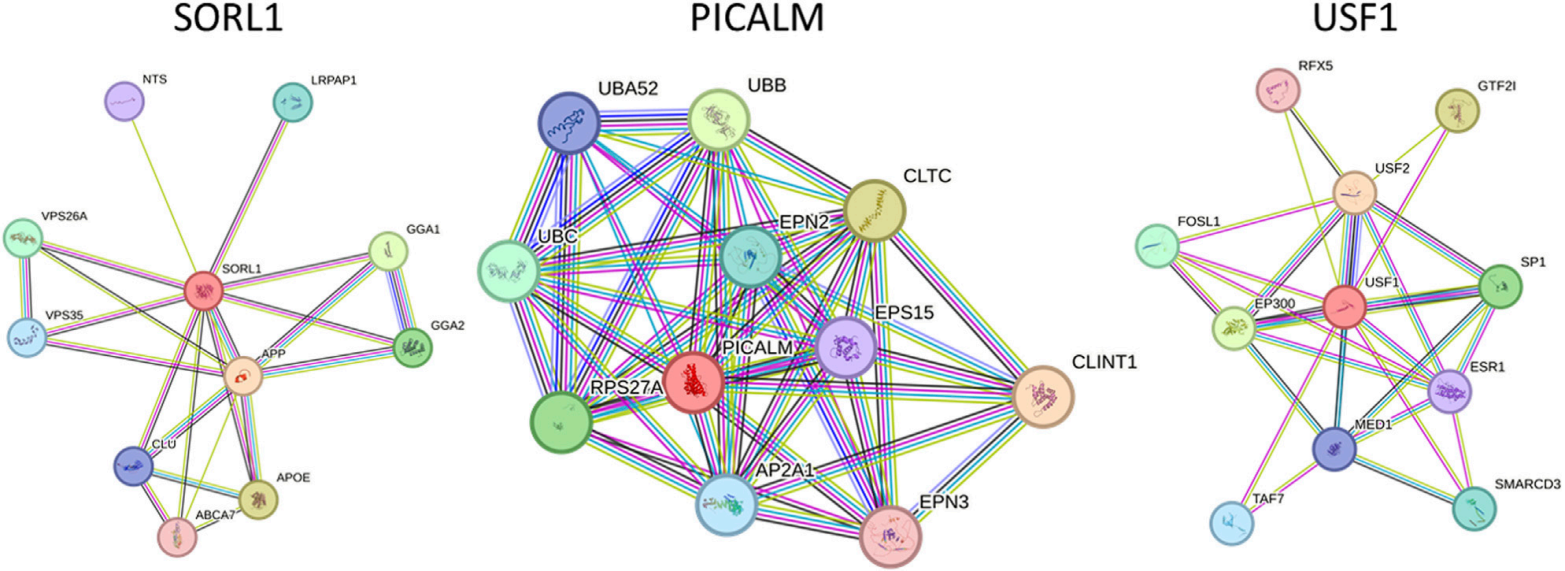
Functional association network of miR-153 target proteins. High-confidence protein-protein interaction network of identified proteins derived from the STRING database. Each protein is represented as a node with edged interactions.

STRING 12 analysis exhibited a strong association (score >0.7) of miR-153 target proteins with various other proteins, i.e., SORL1 exhibited strong interaction with GGA1, GGA2, APOE, ABCA7, CLU, APP, VPS35, VPS26A, LRPAP1, and NTS; PICALM is strongly associated with CLINT1, AP2A1, EPS15, RPS27A, CLTC, EPN2, EPN3, UBA52, UBB and UBC. Similarly, USF1 also showed significant interaction with ten different proteins such as SP1, ESR1, SMARCD3, EP300, FOSL1, USF2, MED1, RFX5, TAF7, and GTF2I (Figure 1). The functions and complete names of all the interacting partners are listed in Table 2.

**TABLE 2:** Functional association of SORL1, PICALM, and USF1 along with interacting partner derived from the STRING database.

Protein	Interacting partner	Function	Score
SORL1	APOE (Apolipoprotein E)	A protein associated with lipid particles, that mainly functions in lipoprotein-mediated lipid transport between organs via the plasma and interstitial fluids	0.997
	ABCA7 (Phospholipid-transporting ATPase ABCA7)	Catalyzes the translocation of specific phospholipids from the cytoplasmic to the extracellular/lumenal leaflet of membrane coupled to the hydrolysis of ATP	0.841
	APP (Amyloid precursor protein)	Functions as a cell surface receptor and performs physiological functions on the surface of neurons relevant to neurite growth, neuronal adhesion, and axonogenesis	0.999
	GGA1 (Golgi-associated, gamma adaptin ear containing, ARF binding protein 1)	Plays a role in protein sorting and trafficking between the trans-Golgi network (TGN) and endosomes	0.986
	GGA2 (ADP-ribosylation factor-binding protein GGA2)	Mediates the ARF-dependent recruitment of clathrin to the TGN and binds ubiquitinated proteins and membrane cargo molecules with a cytosolic acidic cluster-dileucine (DXXLL) motif	0.951
	VPS26A (Vacuolar protein sorting 26 homolog A)	Acts as a component of the retromer cargo-selective complex (CSC)	0.946
	VPS35 (Vacuolar protein sorting 35 homologs)	Acts as a component of the retromer cargo-selective complex (CSC). The CSC prevents the mis-sorting of selected transmembrane cargo proteins into the lysosomal degradation pathway	0.877
	CLU (Clusterin alpha chain; [Isoform 1])	Functions as an extracellular chaperone that prevents aggregation of non-native proteins	0.862
	LRPAP1 (low-density lipoprotein receptor-related protein associated protein 1)	Molecular chaperone for LDL receptor-related proteins that may regulate their ligand binding activity along the secretory pathway	0.911
	NTS (Neurotensin/neuromedin N)	Neurotensin may play an endocrine or paracrine role in the regulation of fat metabolism	0.852
PICALM	CLINT1 (Clathrin interactor 1)	May have a role in transport via clathrin-coated vesicles from the trans-Golgi network to endosomes	0.982
	RPS27A (biquitin-40S ribosomal protein S27a)	It exists in independent form or is attached to other proteins to modify their functions	0.949
	EPN2 (Epsin-2)	Plays a role in the formation of clathrin-coated invaginations and endocytosis	0.947
	CTLC (clathrin, heavy chain 1)	Clathrin is the major protein of the polyhedral coat of coated pits and vesicles	0.971
	EPN3 (Epsin-3)	Mediates apoptosis	0.944
	AP2A1 (Adaptor-related protein complex 2, alpha 1 subunit)	Adaptor protein complexes function in protein transport via transport vesicles in different membrane traffic pathways	0.947
	UBA52 (biquitin-60S ribosomal protein L40)	It is a component of 60S ribosomal subunit	0.947
	UBB (Polyubiquitin-B)	It exists in independent form or is attached to other proteins to modify their functions	0.950
	UBC (Ubiquitin C)	It exists in independent form or is attached to other proteins to modify their functions	0.949
	EPS15 (Epidermal growth factor receptor pathway substrate 15)	Involved in cell growth regulation	0.946
USF1	SP1 (Transcription factor Sp1)	It can activate or repress transcription in response to physiological and pathological signals	0.901
	ESR1 (estrogen receptor 1)	Involved in the regulation of eukaryotic gene expression and affect cellular proliferation and differentiation in target tissues	0.796
	SMARCD3 (SWI/SNF-related matrix-associated actin-dependent regulator of chromatin subfamily D member 3)	Stimulates nuclear receptor mediated transcription	0.819
	EP300 (Histone acetyltransferase p300)	Functions as histone acetyltransferase and regulates transcription via chromatin remodeling	0.912
	FOSL1 (Fos-related antigen 1)	Modulates cellular transformation, multiplication, and differentiation	0.846
	USF2 (Upstream stimulatory factor 2)	Transcription factor that binds to a symmetrical DNA sequence (E-boxes)	0.999
	MED1(Mediator of RNA polymerase II transcription subunit 1)	A coactivator involved in the regulated transcription of nearly all RNA polymerase II-dependent genes	0.801
	RFX5 (NA-binding protein RFX5)	Activates transcription from class II MHC promoters	0.752
	TAF7 (Transcription initiation factor TFIID subunit 7)	Functions as a component of the DNA-binding general transcription factor complex TFIID.	0.814
	GTF2I (General transcription factor II-I)	Acts as a coregulator for USF1 by binding independently two promoter elements, a pyrimidine-rich initiator (Inr) and an upstream E-box	0.958

### 3.3 Functional enrichment and plausible port translational modifications analysis

The identified miR-153 target proteins were functionally annotated using WEBGESTALT and Uniprot (www.uniprot.org/). Target proteins were classified based on molecular function, biological process, and cellular localization (Table 3). All three proteins are actively involved in different biological processes. SORL1 is a neuronal apolipoprotein E receptor and its gene is predominantly expressed in the central nervous system (CNS) and is involved in beta-amyloid binding, vesicle-mediated transport, cholesterol metabolic process, negative regulation of neurogenesis, and various other important cellular pathways. PICALM plays an important role in clathrin-mediated endocytosis, vesicle-mediated transport, axonogenesis, neuron projection development, neuronal differentiation, dendrite development, and many other different processes. USF1 acts as a transcription factor and belongs to the basic helix-loop-helix leucine zipper family which is known to regulate the macromolecule metabolic process, cellular metabolic process, coagulation, hypoxia, glucose homeostasis, fibrinolysis, and nutrient levels.

**TABLE 3:** Functional distribution of SORL1, PICALM and USF1 on the basis of biological process, molecular function and cellular compartment.

	SORL1	PICALM	USF1
Biological processes	Vesicle mediated transport	Vesicle mediated transport Endocytosis Receptor mediated endocytosis Plasma membrane part Positive regulation of macromolecule metabolic process Positive regulation of cellular metabolic process Receptor metabolic process Cell part morphogenesis Cell projection morphogenesis Neuron projection development Axonogenesis Cell morphogenesis involved in neuronal differentiation Dendrite development Synapse	Positive regulation of macromolecule metabolic process
	Sterol metabolic process		Positive regulation of cellular metabolic process
	Cholesterol metabolic process		Cellular response to nutrient levels
	Negative regulation of neurogenesis		Coagulation
	Negative regulation of beta-amyloid formation		Response to hypoxia
	Positive regulation of protein catabolic process		Glucose homeostasis Negative regulation to fibrinolysis
Molecular function	Beta-Amyloid binding	Phosphatidylinositol-4,5-bisphosphate 5 phosphatase activity	Transcription factor binding
	Protein transporter activity		MAP Kinase activity
	Beta-aspartyl-transferase activity		
Cellular Components	Early endosome Endoplasmic reticulum Extracellular exosome	Golgi apparatus Clathrin coated vesicles	Nucleoplasm
	Membrane	Neurofibrillary tangle	Nucleus
	Nuclear envelop Lumen	Neuronal cell body Pre and post synaptic membrane	Transcription factor complex
MicroRNA Targets	MIR-17–5p, MIR-20A, MIR-106A, MIR106B, MIR-20B and MIR-519D	MIR-520F	

A total of 49 serine, tyrosine, and threonine sites were predicted as plausible phosphorylation sites for USF1, 29 for PICALM, and 368 sites for SORL1. The *S*-nitrosylation prediction analysis revealed 2 cysteine residue sites at positions 229 and 248 for USF1, while 3 and 4 sites were predicted for PICALM and SORL1, respectively. The cysteine modifications for PICALM were predicted at positions 27, 48, and 230 whereas positions 942, 1,042, 1,502, and 1,593 of SORL1 are susceptible to cysteine modifications. The N and O glycosylation analysis for the target proteins also showed significant susceptibility for these PTMs. A total of 6 sites were predicted as plausible sites for N-glycosylation in the PICALM sequence at positions 69, 105, 384, 445, 505, and 513. The O-glycosylation was also predicted for 58 sites of PICALM For USF1, 43 sites were predicted for O-glycosylation while no plausible sites were identified for N-glycosylation. The SORL1 has 28 predicted sites for N-glycosylation while 314 sites were found to be susceptible to O-glycosylation (Supplementary Data).

## 4 Discussion

By regulating the expression of target genes, miRNAs mediate various biological processes. Different miRNAs are reported to associate with AD, however, miR-153 plays a crucial role in regulating the expression of amyloid precursor protein (APP). Its expression is significantly downregulated in early and late-stage AD as observed in the APPswe/PSΔE9 murine model (Liang et al., 2012). SNHG1-mediated suppression of miR-153 increases neurotoxicity in SH-SY5Y cells (Zhao et al., 2020). Inversely, increased expression of miR-153 protects the neurons from cellular death via the upregulation of PRX5 (Xu et al., 2019). Similarly, miR-153–3p reduces LPS-induced neuroinflammation and subsequently cell death by inhibiting the NF-κB signaling pathway (Choi et al., 2022).

miR-153 obstructs APP production in neurons therefore its deregulation may drive over-expression of APP and subsequently leads to AD progression. Apart from APP miR-153 also reduced the expression of APLP2, an (APP homolog), in human fetal brain cultures therefore, it was hypothesized that it may target some of the other critical genes linked to neurodegeneration and AD development (Long et al., 2012). In this study, five main culprits of AD pathogenesis were found to be negatively regulated by miR-153 that include; APP, SORL1, PICALM, USF1, and PSEN1. The relationship between miR-153 and APP expression is well established while PSEN1 is predicted by just one algorithm hence we primarily focused on SORL1, PICALM, and USF1.

Apart from the direct role of these genes in AD, the complex interaction with various important disease-promoting/alleviating entities is revealed by the STRING database. The interaction network exhibits that complex multi-dimensional regulation takes place between key AD players, such as APP, SORL1, PICALM, USF1, PSEN1, and other disease-causing agents. The predicted genes/proteins are significant to neuroprotection, synapse formation, memory and learning, intellectual abilities, and neurodegeneration (Chandrasekaran and Bonchev, 2016). Neuronal sortilin receptor-related gene (SORL1) mediates the intracellular trafficking of APP and dysregulation of the particular process leads to Aβ accumulation and subsequently neuronal apoptosis. The exact underlying mechanisms determining the influence of SORL1 on APP trafficking and export are not explicitly studied therefore opening new avenues to investigate AD from a different perspective (Lee et al., 2008). SORL1 exhibited strong interaction with various proteins, such as GGA1, GGA2, APOE, ABCA7, CLU, APP, VPS35, VPS26A, LRPAP1, and NTS. Apolipoprotein E (APOE) modulates lipid metabolism and is implicated in AD pathogenesis. Lower levels of APOE are linked with a decline in cognitive abilities. Genetic variations in the APOE region alter the plasma expression levels of this gene and increase the risk for AD (Aslam et al., 2023). APOE ε4 allele leads to poor cognitive abilities and increased amyloid beta burden in the brain. Moreover, it alters the microglial immune response by downregulating innate immunity (lysosomal and complement pathways) and inducing stress-like responses (Liu et al., 2023). Apolipoproteins mediate cholesterol metabolism mainly via ABCA1 (ATP-binding cassette transporter A1) (Chen et al., 2013). ABCA1 is widely present in neurons and astrocytes and maintains cholesterol homeostasis in the brain. A recent study reported that amyloid beta-mediated dysfunctional ABCA1 in astrocytes altered the transport of cholesterol from astrocytes to the neurons. It subsequently led to impairment of cholesterol metabolism, a prominent feature of AD pathogenesis (Azizidoost et al., 2022). Clusterin (CLU) plays a protective role in the brain however, mutations in CLU increase the risk of developing AD. The rs11136000C mutation in CLU causes dysregulation in GABAergic signaling thus promoting AD pathogenesis (Chen et al., 2023).

Phosphatidylinositol-binding clathrin assembly protein (PICALM) is associated with clathrin-mediated endocytosis (Kyriazis et al., 2008). It is predominantly situated in neurons, oligodendrocytes, astrocytes, and endothelial cells where it recruits the adaptor protein 2 (AP-2) and clathrin to the plasma membrane to encapsulate the target proteins (Yao et al., 2003). The clathrin-coated vesicles are further processed in endosomes or lysosomes to be removed from the cell. PICALM is also associated with the removal of Aβ from the cells, therefore, minimizing the plaque burden and preventing AD pathology. Altered PICALM expression levels are reported in AD brain tissues however, it is yet to be determined whether it affects the Aβ transport or is influenced by Aβ levels (Baig et al., 2010). PICALM is strongly associated with various other proteins and alterations in its expression may influence the biological activities of target proteins correspondingly. The interacting partners of PICALM include; CLINT1, AP2A1, EPS15, RPS27A, CLTC, EPN2, EPN3, UBA52, UBB and UBC. RPS27A is a fusion protein consisting of ubiquitin and S27a (ribosomal protein) (Sayers et al., 2018). An *in silico* analysis revealed the potential role of RPS27A in neurodegenerative disorders by modulating the expression of Il-18 and Cx3cl1 (Khayer et al., 2020). The role of other target proteins is still unclear in AD and needs further research.

Upstream transcription factor 1 (USF1), a ubiquitously expressed gene encodes a transcription factor that stimulates the transcription of various lipid and glucose-metabolizing genes (Lee et al., 2006) including APOE (Salero et al., 2003). USF1 plays a significant role in abnormal lipid aggregation (Guo et al., 2018), neuronal differentiation, and synaptic plasticity, moreover activates the APP promoter thereby affecting Aβ production and processing (Isotalo et al., 2012). USF1 strongly interacts with various other proteins such as SP1, ESR1, SMARCD3, EP300, FOSL1, USF2, MED1, RFX5, TAF7, and GTF2I. ESR1 (Estrogen receptor 1) is implicated in AD progression and it is described that ESR1 mutant (rs9340803) may lead to AD by perturbing cholesterol metabolism and accumulating amyloid beta in the brain. Nevertheless, further studies on larger cohorts are required to confirm the role of the ESR1 variant in AD (Li et al., 2018).

The post-translational modification data for the target proteins revealed a significant number of predicted sites with susceptibility towards phosphorylation, S-nitrosylation, and N and O-glycosylation. There is ample evidence that PTMs play a crucial role in AD pathology (Marcelli et al., 2018). Phosphorylation of tau and amyloid beta is detected in AD mouse models and these modifications affect the functions of microtubules and synapses, respectively (Wang et al., 2023).

Identification and validation of these predicted PTM sites and their pathological correlation with miR-153 targets will also provide substantial data that will be helpful in further elucidation of molecular mechanisms involved in AD pathology.

In this study, bioinformatics analysis predicted some of the important AD-related targets of miR-153. The gene ontology (GO) analysis of putative miR-153 targets revealed their important functions relevant to AD such as regulation of Aβ formation, negative regulation of neurogenesis, neuronal projection development, synapse formation, and NFTs formation. miRNAs perform their regulatory functions by affecting the target genes therefore it is crucial to study the potential targets and their underlying effects. This approach will facilitate the identification of novel regulatory networks of various miRNAs in different disease-related processes.

## 5 Conclusion

Our findings may aid the understanding of different molecular mechanisms and identification of effective therapeutic targets for AD. Further experimental studies may provide additional insights into the regulatory role of miR-153 and its targets in the development of AD and other neurodegenerative disorders.

## Data Availability

The datasets presented in this study can be found in online repositories. The names of the repository/repositories and accession number(s) can be found in the article/Supplementary Material.

## References

[B1] Amber S. Mirza F. J. Asif M. Hassan D. Ahmed T. Zahid S. (2020). Amyloid-beta induced neurotoxicity impairs cognition and adult hippocampal neurogenesis in a mouse model for Alzheimer’s disease. Curr. Alzheimer Res. 17 (11), 1033–1042. 10.2174/1567205017666201224162730 33357181

[B2] Arshad M. Bhatti A. John P. (2018). Identification and *in silico* analysis of functional SNPs of human TAGAP protein: a comprehensive study. PloS one 13 (1), e0188143. 10.1371/journal.pone.0188143 29329296 PMC5766082

[B3] Aslam M. M. Fan K. H. Lawrence E. Bedison M. A. Snitz B. E. DeKosky S. T. (2023). Genome-wide analysis identifies novel loci influencing plasma apolipoprotein E concentration and Alzheimer’s disease risk. Mol. Psychiatry 5, 1–2. 10.1038/s41380-023-02170-4 PMC10827653 37666928

[B4] Azevedo R. Silva A. M. N. Reis C. A. Santos L. L. Ferreira J. A. (2018). *In silico* approaches for unveiling novel glycobiomarkers in cancer. J. Proteomics 171, 95–106. 10.1016/j.jprot.2017.08.004 28782717

[B5] Azizidoost S. Babaahmadi-Rezaei H. Nazeri Z. Cheraghzadeh M. Kheirollah A. (2022). Amyloid beta increases ABCA1 and HMGCR protein expression, and cholesterol synthesis and accumulation in mice neurons and astrocytes. Biochimica Biophysica Acta (BBA)-Molecular Cell Biol. Lipids 1867 (1), 159069. 10.1016/j.bbalip.2021.159069 34744007

[B6] Baig S. Joseph S. Tayler H. Abraham R. Owen M. Williams J. (2010). Distribution and expression of picalm in Alzheimer disease. J. Neuropathol. Exp. Neurol. 69, 1071–1077. 10.1097/NEN.0b013e3181f52e01 20838239 PMC3017341

[B7] Calin G. A. Dumitru C. D. Shimizu M. Bichi R. Zupo S. Noch E. (2002). Frequent deletions and down-regulation of micro-RNA genes miR15 and miR16 at 13q14 in chronic lymphocytic leukemia. Proc. Natl. Acad. Sci. 99, 15524–15529. 10.1073/pnas.242606799 12434020 PMC137750

[B8] Carr G. Barrese V. Stott J. B. Povstyan O. V. Jepps T. A. Figueiredo H. B. (2016). MicroRNA-153 targeting of KCNQ4 contributes to vascular dysfunction in hypertension. Cardiovasc Res. 112, 581–589. 10.1093/cvr/cvw177 27389411 PMC5079273

[B9] Chandrasekaran S. Bonchev D. (2016). Network topology analysis of post-mortem brain microarrays identifies more alzheimer’s related genes and micrornas and points to novel routes for fighting with the disease. PloS One 11, e0144052. 10.1371/journal.pone.0144052 26784894 PMC4718516

[B10] Chen C. Tang X. Lan Z. Chen W. Su H. Li W. (2023). GABAergic signaling abnormalities in a novel CLU mutation Alzheimer's disease mouse model. Transl. Res. 260, 32–45. 10.1016/j.trsl.2023.05.003 37211336

[B11] Chen J. Zhang X. Kusumo H. Costa L. G. Guizzetti M. (2013). Cholesterol efflux is differentially regulated in neurons and astrocytes: implications for brain cholesterol homeostasis. Biochimica Biophysica Acta (BBA)-Molecular Cell Biol. Lipids 1831 (2), 263–275. 10.1016/j.bbalip.2012.09.007 PMC3534809 23010475

[B12] Choi H. R. Ha J. S. Kim E. A. Cho S. W. Yang S. J. (2022). MiR-30a-5p and miR-153-3p regulate LPS-induced neuroinflammatory response and neuronal apoptosis by targeting NeuroD1. BMB Rep. 55 (9), 447–452. 10.5483/BMBRep.2022.55.9.061 35651331 PMC9537026

[B13] Dong X. Wang H. Zhan L. Li Q. Li Y. Wu G. (2023). miR-153-3p suppresses the differentiation and proliferation of neural stem cells via targeting GPR55. Aging (Albany NY) 15 (16), 8518–8527. 10.18632/aging.204002 37642951 PMC10497013

[B14] Femminella G. D. Ferrara N. Rengo G. (2015). The emerging role of micrornas in alzheimer's disease. Front. Physiol. 6, 40. 10.3389/fphys.2015.00040 25729367 PMC4325581

[B15] Filipowicz W. Bhattacharyya S. N. Sonenberg N. (2008). Mechanisms of post-transcriptional regulation by micrornas: are the answers in sight? Nat. Rev. Genet. 9, 102–114. 10.1038/nrg2290 18197166

[B16] François M. Bull C. F. Fenech M. F. Leifert W. R. (2019). Current state of saliva biomarkers for aging and alzheimer's disease. Curr. Alzheimer Res. 16, 56–66. 10.2174/1567205015666181022094924 30345919

[B17] Guo J. Fang W. Chen X. Lin Y. Hu G. Wei J. (2018). Upstream stimulating factor 1 suppresses autophagy and hepatic lipid droplet catabolism by activating mTOR. FEBS Lett. 592 (16), 2725–2738. 10.1002/1873-3468.13203 30054905 PMC6175420

[B18] Hamzeiy H. Allmer J. Yousef M. (2014). “Computational methods for MicroRNA target prediction,” in miRNomics: MicroRNA biology and computational analysis. Methods in molecular biology (methods and protocols). Editors Yousef M. Allmer J. (Totowa, NJ: Humana Press), 207–221. 10.1007/978-1-62703-748-8_12 24272439

[B19] Huang J. Weng Q. Shi Y. Mao W. Zhao Z. Wu R. (2020). MicroRNA‐155‐5p suppresses PD‐L1 expression in lung adenocarcinoma. FEBS Open Bio 10 (6), 1065–1071. 10.1002/2211-5463.12853 PMC7262882 32237066

[B20] Isotalo K. Kok E. H. Luoto T. M. Haikonen S. Haapasalo H. Lehtimäki T. (2012). Upstream transcription factor 1 (USF1) polymorphisms associate with Alzheimer's disease‐related neuropathological lesions: tampere Autopsy Study. Brain Pathol. 22, 765–775. 10.1111/j.1750-3639.2012.00586.x 22390463 PMC8057645

[B21] Jaberi K. R. Alamdari-Palangi V. Jaberi A. R. Esmaeli Z. Shakeri A. Hayat S. M. (2024). The regulation, functions, and signaling of miR-153 in neurological disorders, and its potential as a biomarker and therapeutic target. Curr. Mol. Med. 23 (9), 863–875. 10.2174/1566524023666220817145638 35980063

[B22] Khayer N. Mirzaie M. Marashi S. A. Jalessi M. (2020). Rps27a might act as a controller of microglia activation in triggering neurodegenerative diseases. Plos one 15 (9), e0239219. 10.1371/journal.pone.0239219 32941527 PMC7498011

[B23] Kwan S. H. Wan-Ibrahim W. I. Juvarajah T. Fung S. Y. Abdul-Rahman P. S. (2021). Isolation and identification of O-and N-linked glycoproteins in milk from different mammalian species and their roles in biological pathways which support infant growth. Electrophoresis 42 (3), 233–244. 10.1002/elps.202000142 33085102

[B24] Kyriazis G. A. Wei Z. Vandermey M. Jo D. G. Xin O. Mattson M. P. (2008). Numb endocytic adapter proteins regulate the transport and processing of the amyloid precursor protein in an isoform-dependent manner implications for alzheimer disease pathogenesis. J. Biol. Chem. 283, 25492–25502. 10.1074/jbc.M802072200 18599481 PMC2533073

[B25] Lee J. C. Lusis A. J. Pajukanta P. (2006). Familial combined hyperlipidemia: upstream transcription factor 1 and beyond. Curr. Opin. Lipidol. 17, 101–109. 10.1097/01.mol.0000217890.54875.13 16531745

[B26] Lee J. H. Barral S. Reitz C. (2008). The neuronal sortilin-related receptor gene sorl1 and late-onset alzheimer’s disease. Curr. Neurol. Neurosci. Rep. 8, 384–391. 10.1007/s11910-008-0060-8 18713574 PMC2694663

[B27] Li X. Zhu X. Zhang W. Yang F. Hui J. Tan J. (2018). The etiological effect of a new low-frequency ESR1 variant on Mild Cognitive Impairment and Alzheimer’s Disease: a population-based study. Aging (Albany NY) 10 (9), 2316–2337. 10.18632/aging.101548 30222591 PMC6188501

[B28] Liang C. Zhu H. Xu Y. Huang L. Ma C. Deng W. (2012). MicroRNA-153 negatively regulates the expression of amyloid precursor protein and amyloid precursor-like protein 2. Brain Res. 1455, 103–113. 10.1016/j.brainres.2011.10.051 22510281

[B29] Liu C. C. Wang N. Chen Y. Inoue Y. Shue F. Ren Y. (2023). Cell-autonomous effects of APOE4 in restricting microglial response in brain homeostasis and Alzheimer’s disease. Nat. Immunol. 19, 1854–1866. 10.1038/s41590-023-01640-9 PMC11980647 37857825

[B30] Long J. M. Ray B. Lahiri D. K. (2012). MicroRNA-153 physiologically inhibits expression of amyloid-β precursor protein in cultured human fetal brain cells and is dysregulated in a subset of Alzheimer disease patients. J. Biol. Chem. 287, 31298–31310. 10.1074/jbc.M112.366336 22733824 PMC3438960

[B31] Marcelli S. Corbo M. Iannuzzi F. Negri L. Blandini F. Nistico R. (2018). The involvement of post-translational modifications in Alzheimer's disease. Curr. Alzheimer Res. 15, 313–335. 10.2174/1567205014666170505095109 28474569

[B32] Mazina A. Shumilina J. Gazizova N. Repkin E. Frolov A. Minibayeva F. (2023). S-nitrosylated proteins involved in autophagy in *Triticum aestivum* roots: a bottom-up proteomics approach and *in silico* predictive algorithms. Life 13 (10), 2024. 10.3390/life13102024 37895406 PMC10608115

[B33] Oliveira A. C. Bovolenta L. A. Nachtigall P. G. Herkenhoff M. E. Lemke N. Pinhal D. (2017). Combining results from distinct microRNA target prediction tools enhances the performance of analyses. Front. Genet. 16 (8), 59. 10.3389/fgene.2017.00059 PMC5432626 28559915

[B34] Pu M. Chen J. Tao Z. Miao L. Qi X. Wang Y. (2019). Regulatory network of miRNA on its target: coordination between transcriptional and post-transcriptional regulation of gene expression. Cell. Mol. Life Sci. 76, 441–451. 10.1007/s00018-018-2940-7 30374521 PMC11105547

[B35] Qiao J. Zhao J. Chang S. Sun Q. Liu N. Dong J. (2020). MicroRNA-153 improves the neurogenesis of neural stem cells and enhances the cognitive ability of aged mice through the notch signaling pathway. Cell Death Differ. 27 (2), 808–825. 10.1038/s41418-019-0388-4 31296962 PMC7206122

[B36] Salero E. Giménez C. Zafra F. (2003). Identification of a non-canonical E-box motif as a regulatory element in the proximal promoter region of the apolipoprotein E gene. Biochem. J. 370, 979–986. 10.1042/BJ20021142 12444925 PMC1223214

[B37] Sayers E. W. Agarwala R. Bolton E. E. Brister J. R. Canese K. Connor R. (2018). Database resources of the national center for biotechnology information. Nucleic acids Res. 46, D8–D13. 10.1093/nar/gkx1095 29140470 PMC5753372

[B38] Schwarzenbach H. Nishida N. Calin G. A. Pantel K. (2014). Clinical relevance of circulating cell-free microRNAs in cancer. Nat. Rev. Clin. Oncol. 11, 145–156. 10.1038/nrclinonc.2014.5 24492836

[B39] Szklarczyk D. Morris J. H. Cook H. Kuhn M. Wyder S. Simonovic M. (2017). The STRING database in 2017: quality-controlled protein-protein association networks, made broadly accessible. Nucleic Acids Res. 45, D362–D368. 10.1093/nar/gkw937 27924014 PMC5210637

[B40] Vejnar C. E. Zdobnov E. M. (2012). MiRmap: comprehensive prediction of microRNA target repression strength. Nucleic acids Res. 40 (22), 11673–11683. 10.1093/nar/gks901 23034802 PMC3526310

[B41] Wang J. Vasaikar S. Shi Z. Greer M. Zhang B. (2017). WebGestalt 2017: a more comprehensive, powerful, flexible and interactive gene set enrichment analysis toolkit. Nucleic Acids Res. 45, W130–W137. 10.1093/nar/gkx356 28472511 PMC5570149

[B42] Wang Q. Xia C. Zhu A. Bao Y. Lu J. Chen Y. (2023). Discrepancy of synaptic and microtubular protein phosphorylation in the hippocampus of APP/PS1 and MAPT× P301S transgenic mice at the early stage of Alzheimer’s disease. Metab. Brain Dis. 9, 1983–1997. 10.1007/s11011-023-01209-3 37160613

[B43] Wong N. Wang X. (2015). miRDB: an online resource for microRNA target prediction and functional annotations. Nucleic Acids Res. 43, D146–D152. 10.1093/nar/gku1104 25378301 PMC4383922

[B44] Xu C. Wang C. Meng Q. Gu Y. Wang Q. Xu W. (2019). miR-153 promotes neural differentiation in the mouse hippocampal HT-22 cell line and increases the expression of neuron-specific enolase. Mol. Med. Rep. 20 (2), 1725–1735. 10.3892/mmr.2019.10421 31257504 PMC6625396

[B45] Xue W. X. Zhang M. Y. Li R. Liu X. Yin Y. H. Qu Y. Q. (2020). Serum miR-1228-3p and miR-181a-5p as noninvasive biomarkers for non-small cell lung cancer diagnosis and prognosis. BioMed Res. Int. 2020, 1–13. 10.1155/2020/9601876 PMC7364230 32724822

[B46] Yao P. Zhang P. Mattson M. Furukawa K. (2003). Heterogeneity of endocytic proteins: distribution of clathrin adaptor proteins in neurons and glia. Neuroscience 121, 25–37. 10.1016/s0306-4522(03)00431-7 12946697

[B47] Zhang S. Yan M. L. Yang L. An X. B. Zhao H. M. Xia S. N. (2020). MicroRNA-153 impairs hippocampal synaptic vesicle trafficking via downregulation of synapsin I in rats following chronic cerebral hypoperfusion. Exp. Neurol. 332, 113389. 10.1016/j.expneurol.2020.113389 32580014

[B48] Zhao J. Geng L. Chen Y. Wu C. (2020). SNHG1 promotes MPP+ induced cytotoxicity by regulating PTEN/AKT/mTOR signaling pathway in SH-SY5Y cells via sponging miR-153-3p. Biol. Res. 53 (1), 1. 10.1186/s40659-019-0267-y 31907031 PMC6943908

