# Supplementary material for: An in silico approach to identify potential downstream targets of miR-153 involved in Alzheimer’s disease
Source: Front Genet. 2024 Jan 16;15:1271404. doi: 10.3389/fgene.2024.1271404 (PMC10824926; doi:10.3389/fgene.2024.1271404)

**Phosphatidylinositol binding clathrin assembly protein [Homo sapiens] (PICALM)**

>MSGQSLTDRITAAQHSVTGSAVSKTVCKATTHEIMGPKKKHLDYLIQCTNEMNVNIPQLADSLFERTTNS

SWVVVFKSLITTHHLMVYGNERFIQYLASRNTLFNLSNFLDKSGLQGYDMSTFIRRYSRYLNEKAVSYRQ

VAFDFTKVKRGADGVMRTMNTEKLLKTVPIIQNQMDALLDFNVNSNELTNGVINAAFMLLFKDAIRLFAA

YNEGIINLLEKYFDMKKNQCKEGLDIYKKFLTRMTRISEFLKVAEQVGIDRGDIPDLSQAPSSLLDALEQ

HLASLEGKKIKDSTAASRATTLSNAVSSLASTGLSLTKVDEREKQAALEEEQARLKALKEQRLKELAKKP

HTSLTTAASPVSTSAGGIMTAPAIDIFSTPSSSNSTSKLPNDLLDLQQPTFHPSVHPMSTASQVASTWGG

FTPSPVAQPHPSAGLNVDFESVFGNKSTNVIVDSGGFDELGGLLKPTVASQNQNLPVAKLPPSKLVSDDL

DSSLANLVGNLGIGNGTTKNDVNWSQPGEKKLTGGSNWQPKVAPTTAWNAATMNGMHFPQYAPPVMAYPA

TTPTGMIGYGIPPQMGSVPVMTQPTLIYSQPVMRPPNPFGPVSGAQIQFM

>AAH73961.1 610 amino acids

**PICALM Phosphorylation DATA**

# **netphos-3.1b** prediction results

# Sequence # x Context Score Kinase Answer

# -------------------------------------------------------------------

1. # AAH73961.1 16 S AAQHSVTGS 0.974 unsp YES
2. # AAH73961.1 62 S QLADSLFER 0.896 unsp YES
3. # AAH73961.1 128 S IRRYSRYLN 0.994 unsp YES
4. # AAH73961.1 128 S IRRYSRYLN 0.846 PKA YES
5. # AAH73961.1 137 S EKAVSYRQV 0.846 unsp YES
6. # AAH73961.1 161 T RTMNTEKLL 0.888 unsp YES
7. # AAH73961.1 273 S QAPSSLLDA 0.989 unsp YES
8. # AAH73961.1 284 S QHLASLEGK 0.924 unsp YES
9. # AAH73961.1 293 S KIKDSTAAS 0.956 unsp YES
10. # AAH73961.1 301 T SRATTLSNA 0.935 unsp YES
11. # AAH73961.1 355 T HTSLTTAAS 0.859 PKC YES
12. # AAH73961.1 359 S TTAASPVST 0.942 unsp YES
13. # AAH73961.1 362 S ASPVSTSAG 0.922 unsp YES
14. # AAH73961.1 381 S FSTPSSSNS 0.847 unsp YES
15. # AAH73961.1 382 S STPSSSNST 0.878 unsp YES
16. # AAH73961.1 383 S TPSSSNSTS 0.853 unsp YES
17. # AAH73961.1 385 S SSSNSTSKL 0.971 unsp YES
18. # AAH73961.1 387 S SNSTSKLPN 0.801 unsp YES
19. # AAH73961.1 409 S VHPMSTASQ 0.926 unsp YES
20. # AAH73961.1 422 T WGGFTPSPV 0.877 unsp YES
21. # AAH73961.1 562 T YPATTPTGM 0.926 unsp YES


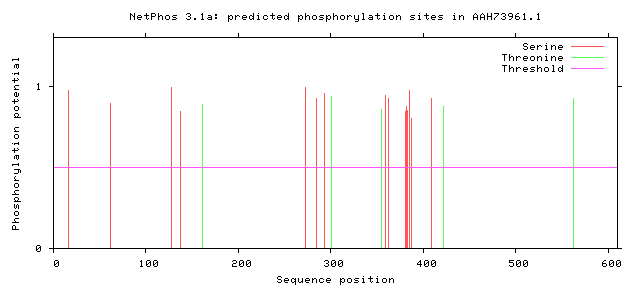


**PICALM N-Glycosylation DATA**

**SeqName Position Potential Jury N-Glyc result**

**agreement**

**----------------------------------------------------------------------**

1. **AAH73961.1 69 NSSW 0.6346 (8/9) +**
2. **AAH73961.1 105 NLSN 0.7172 (9/9) ++**
3. **AAH73961.1 384 NSTS 0.5358 (7/9) +**
4. **AAH73961.1 445 NKST 0.5150 (5/9) +**
5. **AAH73961.1 505 NGTT 0.6101 (8/9) +**
6. **AAH73961.1 513 NWSQ 0.3536 (9/9) --**

**-----------------------------------------------**


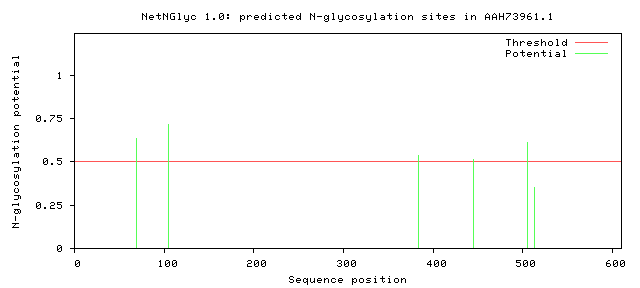


**##source-version NetOGlyc 4.0.0.13 (PICALM)**

#seqname source feature start end score strand frame comment

1. AAH73961_1 netOGlyc-4.0.0.13 CARBOHYD 18 18 0.5 . . #POSITIVE
2. AAH73961_1 netOGlyc-4.0.0.13 CARBOHYD 23 23 0.5962 . . #POSITIVE
3. AAH73961_1 netOGlyc-4.0.0.13 CARBOHYD 30 30 0.628745 . . #POSITIVE
4. AAH73961_1 netOGlyc-4.0.0.13 CARBOHYD 31 31 0.571989 . . #POSITIVE
5. AAH73961_1 netOGlyc-4.0.0.13 CARBOHYD 268 268 0.5 . . #POSITIVE
6. AAH73961_1 netOGlyc-4.0.0.13 CARBOHYD 272 272 0.607312 . . #POSITIVE
7. AAH73961_1 netOGlyc-4.0.0.13 CARBOHYD 273 273 0.597549 . . #POSITIVE
8. AAH73961_1 netOGlyc-4.0.0.13 CARBOHYD 284 284 0.765642 . . #POSITIVE
9. AAH73961_1 netOGlyc-4.0.0.13 CARBOHYD 293 293 0.976127 . . #POSITIVE
10. AAH73961_1 netOGlyc-4.0.0.13 CARBOHYD 294 294 0.922013 . . #POSITIVE
11. AAH73961_1 netOGlyc-4.0.0.13 CARBOHYD 297 297 0.972952 . . #POSITIVE
12. AAH73961_1 netOGlyc-4.0.0.13 CARBOHYD 300 300 0.941315 . . #POSITIVE
13. AAH73961_1 netOGlyc-4.0.0.13 CARBOHYD 301 301 0.948627 . . #POSITIVE
14. AAH73961_1 netOGlyc-4.0.0.13 CARBOHYD 303 303 0.953132 . . #POSITIVE
15. AAH73961_1 netOGlyc-4.0.0.13 CARBOHYD 307 307 0.964871 . . #POSITIVE
16. AAH73961_1 netOGlyc-4.0.0.13 CARBOHYD 308 308 0.973209 . . #POSITIVE
17. AAH73961_1 netOGlyc-4.0.0.13 CARBOHYD 311 311 0.929196 . . #POSITIVE
18. AAH73961_1 netOGlyc-4.0.0.13 CARBOHYD 312 312 0.932238 . . #POSITIVE
19. AAH73961_1 netOGlyc-4.0.0.13 CARBOHYD 315 315 0.873484 . . #POSITIVE
20. AAH73961_1 netOGlyc-4.0.0.13 CARBOHYD 317 317 0.892753 . . #POSITIVE
21. AAH73961_1 netOGlyc-4.0.0.13 CARBOHYD 352 352 0.913678 . . #POSITIVE
22. AAH73961_1 netOGlyc-4.0.0.13 CARBOHYD 353 353 0.916936 . . #POSITIVE
23. AAH73961_1 netOGlyc-4.0.0.13 CARBOHYD 355 355 0.879862 . . #POSITIVE
24. AAH73961_1 netOGlyc-4.0.0.13 CARBOHYD 356 356 0.958357 . . #POSITIVE
25. AAH73961_1 netOGlyc-4.0.0.13 CARBOHYD 359 359 0.972861 . . #POSITIVE
26. AAH73961_1 netOGlyc-4.0.0.13 CARBOHYD 362 362 0.938389 . . #POSITIVE
27. AAH73961_1 netOGlyc-4.0.0.13 CARBOHYD 363 363 0.913928 . . #POSITIVE
28. AAH73961_1 netOGlyc-4.0.0.13 CARBOHYD 364 364 0.842639 . . #POSITIVE
29. AAH73961_1 netOGlyc-4.0.0.13 CARBOHYD 370 370 0.718921 . . #POSITIVE
30. AAH73961_1 netOGlyc-4.0.0.13 CARBOHYD 378 378 0.593654 . . #POSITIVE
31. AAH73961_1 netOGlyc-4.0.0.13 CARBOHYD 379 379 0.687747 . . #POSITIVE
32. AAH73961_1 netOGlyc-4.0.0.13 CARBOHYD 381 381 0.786193 . . #POSITIVE
33. AAH73961_1 netOGlyc-4.0.0.13 CARBOHYD 382 382 0.75359 . . #POSITIVE
34. AAH73961_1 netOGlyc-4.0.0.13 CARBOHYD 383 383 0.65903 . . #POSITIVE
35. AAH73961_1 netOGlyc-4.0.0.13 CARBOHYD 385 385 0.686518 . . #POSITIVE
36. AAH73961_1 netOGlyc-4.0.0.13 CARBOHYD 387 387 0.595248 . . #POSITIVE
37. AAH73961_1 netOGlyc-4.0.0.13 CARBOHYD 400 400 0.694142 . . #POSITIVE
38. AAH73961_1 netOGlyc-4.0.0.13 CARBOHYD 404 404 0.834073 . . #POSITIVE
39. AAH73961_1 netOGlyc-4.0.0.13 CARBOHYD 409 409 0.746122 . . #POSITIVE
40. AAH73961_1 netOGlyc-4.0.0.13 CARBOHYD 410 410 0.824846 . . #POSITIVE
41. AAH73961_1 netOGlyc-4.0.0.13 CARBOHYD 412 412 0.701308 . . #POSITIVE
42. AAH73961_1 netOGlyc-4.0.0.13 CARBOHYD 416 416 0.841406 . . #POSITIVE
43. AAH73961_1 netOGlyc-4.0.0.13 CARBOHYD 417 417 0.707469 . . #POSITIVE
44. AAH73961_1 netOGlyc-4.0.0.13 CARBOHYD 422 422 0.803675 . . #POSITIVE
45. AAH73961_1 netOGlyc-4.0.0.13 CARBOHYD 424 424 0.712213 . . #POSITIVE
46. AAH73961_1 netOGlyc-4.0.0.13 CARBOHYD 432 432 0.652502 . . #POSITIVE
47. AAH73961_1 netOGlyc-4.0.0.13 CARBOHYD 467 467 0.583216 . . #POSITIVE
48. AAH73961_1 netOGlyc-4.0.0.13 CARBOHYD 483 483 0.559411 . . #POSITIVE
49. AAH73961_1 netOGlyc-4.0.0.13 CARBOHYD 508 508 0.544908 . . #POSITIVE
50. AAH73961_1 netOGlyc-4.0.0.13 CARBOHYD 515 515 0.639257 . . #POSITIVE
51. AAH73961_1 netOGlyc-4.0.0.13 CARBOHYD 526 526 0.568591 . . #POSITIVE
52. AAH73961_1 netOGlyc-4.0.0.13 CARBOHYD 535 535 0.816681 . . #POSITIVE
53. AAH73961_1 netOGlyc-4.0.0.13 CARBOHYD 542 542 0.719216 . . #POSITIVE
54. AAH73961_1 netOGlyc-4.0.0.13 CARBOHYD 561 561 0.655355 . . #POSITIVE
55. AAH73961_1 netOGlyc-4.0.0.13 CARBOHYD 562 562 0.602428 . . #POSITIVE
56. AAH73961_1 netOGlyc-4.0.0.13 CARBOHYD 564 564 0.522292 . . #POSITIVE
57. AAH73961_1 netOGlyc-4.0.0.13 CARBOHYD 582 582 0.773943 . . #POSITIVE
58. AAH73961_1 netOGlyc-4.0.0.13 CARBOHYD 589 589 0.867147 . . #POSITIVE

**PICALM S-nitrosylation (GPS-SNO) Data**

27 SAVSKTVCKATTHEI 1.24 0 Cluster A

48 HLDYLIQCTNEMNVN 2.288 0 Cluster B

230 FDMKKNQCKEGLDIY 0.568 0 Cluster A

**>sp|Q92673|SORL_HUMAN Sortilin-related receptor OS=Homo sapiens OX=9606 GN=SORL1 PE=1 SV=2**

MATRSSRRESRLPFLFTLVALLPPGALCEVWTQRLHGGSAPLPQDRGFLVVQGDPRELRL

WARGDARGASRADEKPLRRKRSAALQPEPIKVYGQVSLNDSHNQMVVHWAGEKSNVIVAL

ARDSLALARPKSSDVYVSYDYGKSFKKISDKLNFGLGNRSEAVIAQFYHSPADNKRYIFA

DAYAQYLWITFDFCNTLQGFSIPFRAADLLLHSKASNLLLGFDRSHPNKQLWKSDDFGQT

WIMIQEHVKSFSWGIDPYDKPNTIYIERHEPSGYSTVFRSTDFFQSRENQEVILEEVRDF

QLRDKYMFATKVVHLLGSEQQSSVQLWVSFGRKPMRAAQFVTRHPINEYYIADASEDQVF

VCVSHSNNRTNLYISEAEGLKFSLSLENVLYYSPGGAGSDTLVRYFANEPFADFHRVEGL

QGVYIATLINGSMNEENMRSVITFDKGGTWEFLQAPAFTGYGEKINCELSQGCSLHLAQR

LSQLLNLQLRRMPILSKESAPGLIIATGSVGKNLASKTNVYISSSAGARWREALPGPHYY

TWGDHGGIITAIAQGMETNELKYSTNEGETWKTFIFSEKPVFVYGLLTEPGEKSTVFTIF

GSNKENVHSWLILQVNATDALGVPCTENDYKLWSPSDERGNECLLGHKTVFKRRTPHATC

FNGEDFDRPVVVSNCSCTREDYECDFGFKMSEDLSLEVCVPDPEFSGKSYSPPVPCPVGS

TYRRTRGYRKISGDTCSGGDVEARLEGELVPCPLAEENEFILYAVRKSIYRYDLASGATE

QLPLTGLRAAVALDFDYEHNCLYWSDLALDVIQRLCLNGSTGQEVIINSGLETVEALAFE

PLSQLLYWVDAGFKKIEVANPDGDFRLTIVNSSVLDRPRALVLVPQEGVMFWTDWGDLKP

GIYRSNMDGSAAYHLVSEDVKWPNGISVDDQWIYWTDAYLECIERITFSGQQRSVILDNL

PHPYAIAVFKNEIYWDDWSQLSIFRASKYSGSQMEILANQLTGLMDMKIFYKGKNTGSNA

CVPRPCSLLCLPKANNSRSCRCPEDVSSSVLPSGDLMCDCPQGYQLKNNTCVKQENTCLR

NQYRCSNGNCINSIWWCDFDNDCGDMSDERNCPTTICDLDTQFRCQESGTCIPLSYKCDL

EDDCGDNSDESHCEMHQCRSDEYNCSSGMCIRSSWVCDGDNDCRDWSDEANCTAIYHTCE

ASNFQCRNGHCIPQRWACDGDTDCQDGSDEDPVNCEKKCNGFRCPNGTCIPSSKHCDGLR

DCSDGSDEQHCEPLCTHFMDFVCKNRQQCLFHSMVCDGIIQCRDGSDEDAAFAGCSQDPE

FHKVCDEFGFQCQNGVCISLIWKCDGMDDCGDYSDEANCENPTEAPNCSRYFQFRCENGH

CIPNRWKCDRENDCGDWSDEKDCGDSHILPFSTPGPSTCLPNYYRCSSGTCVMDTWVCDG

YRDCADGSDEEACPLLANVTAASTPTQLGRCDRFEFECHQPKTCIPNWKRCDGHQDCQDG

RDEANCPTHSTLTCMSREFQCEDGEACIVLSERCDGFLDCSDESDEKACSDELTVYKVQN

LQWTADFSGDVTLTWMRPKKMPSASCVYNVYYRVVGESIWKTLETHSNKTNTVLKVLKPD

TTYQVKVQVQCLSKAHNTNDFVTLRTPEGLPDAPRNLQLSLPREAEGVIVGHWAPPIHTH

GLIREYIVEYSRSGSKMWASQRAASNFTEIKNLLVNTLYTVRVAAVTSRGIGNWSDSKSI

TTIKGKVIPPPDIHIDSYGENYLSFTLTMESDIKVNGYVVNLFWAFDTHKQERRTLNFRG

SILSHKVGNLTAHTSYEISAWAKTDLGDSPLAFEHVMTRGVRPPAPSLKAKAINQTAVEC

TWTGPRNVVYGIFYATSFLDLYRNPKSLTTSLHNKTVIVSKDEQYLFLVRVVVPYQGPSS

DYVVVKMIPDSRLPPRHLHVVHTGKTSVVIKWESPYDSPDQDLLYAVAVKDLIRKTDRSY

KVKSRNSTVEYTLNKLEPGGKYHIIVQLGNMSKDSSIKITTVSLSAPDALKIITENDHVL

LFWKSLALKEKHFNESRGYEIHMFDSAMNITAYLGNTTDNFFKISNLKMGHNYTFTVQAR

CLFGNQICGEPAILLYDELGSGADASATQAARSTDVAAVVVPILFLILLSLGVGFAILYT

KHRRLQSSFTAFANSHYSSRLGSAIFSSGDDLGEDDEDAPMITGFSDDVPMVIA

**S-Nitrosylation SORL1**

28 LLPPGALCEVWTQRL 15.62 0 Cluster C

194 LWITFDFCNTLQGFS 0.82 0 Cluster A

362 SEDQVFVCVSHSNNR 1.799 0 Cluster B

467 GYGEKINCELSQGCS 19.642 0 Cluster C

473 NCELSQGCSLHLAQR 20.007 0 Cluster C

625 TDALGVPCTENDYKL 0.366 0 Cluster A

643 SDERGNECLLGHKTV 20.745 0 Cluster C

660 RRTPHATCFNGEDFD 0.284 0 Cluster A

675 RPVVVSNCSCTREDY 0.689 0 Cluster A

677 VVVSNCSCTREDYEC 1.457 0 Cluster B

684 CTREDYECDFGFKMS 0.098 0 Cluster A

699 EDLSLEVCVPDPEFS 17.46 0 Cluster C

716 SYSPPVPCPVGSTYR 20.445 0 Cluster C

736 RKISGDTCSGGDVEA 0.268 0 Cluster A

752 LEGELVPCPLAEENE 17.066 0 Cluster C

801 DFDYEHNCLYWSDLA 0.661 0 Cluster A

816 LDVIQRLCLNGSTGQ 0.579 0 Cluster A

942 WTDAYLECIERITFS 2.668 0 Cluster B

1021 KNTGSNACVPRPCSL 1.375 0 Cluster B

1026 NACVPRPCSLLCLPK 19.599 0 Cluster C

1030 PRPCSLLCLPKANNS 2.185 0 Cluster B

1040 KANNSRSCRCPEDVS 0.443 0 Cluster A

1042 NNSRSCRCPEDVSSS 2.717 0 Cluster B

1058 LPSGDLMCDCPQGYQ 1.043 0 Cluster B

1060 SGDLMCDCPQGYQLK 0.869 0 Cluster A

1071 YQLKNNTCVKQATTI 0.388 0 Cluster A

1079 VKQATTICDLDTQFR 17.555 0 Cluster C

1087 DLDTQFRCQESGTCI 2.402 0 Cluster B

1093 RCQESGTCIPLSYKC 15.161 0 Cluster C

1100 CIPLSYKCDLEDDCG 17.62 0 Cluster C

1106 KCDLEDDCGDNSDES 0.76 0 Cluster A

1115 DNSDESHCEMHQCRS 18.679 0 Cluster C

1120 SHCEMHQCRSDEYNC 0.728 0 Cluster B

1127 CRSDEYNCSSGMCIR 1.707 0 Cluster B

1132 YNCSSGMCIRSSWVC 0.766 0 Cluster B

1139 CIRSSWVCDGDNDCR 0.668 0 Cluster B

1145 VCDGDNDCRDWSDEA 0.246 0 Cluster A

1154 DWSDEANCTAIYHTC 0.377 0 Cluster A

1161 CTAIYHTCEASNFQC 0.164 0 Cluster A

1168 CEASNFQCRNGHCIP 0.23 0 Cluster A

1173 FQCRNGHCIPQRWAC 0.239 0 Cluster B

1180 CIPQRWACDGDTDCQ 0.984 0 Cluster B

1186 ACDGDTDCQDGSDED 1.027 0 Cluster B

1197 SDEDPVNCEKKCNGF 1.332 0 Cluster B

1201 PVNCEKKCNGFRCPN 0.685 0 Cluster B

1206 KKCNGFRCPNGTCIP 0.164 0 Cluster A

1211 FRCPNGTCIPSSKHC 1.152 0 Cluster B

1218 CIPSSKHCDGLRDCS 0.459 0 Cluster A

1224 HCDGLRDCSDGSDEQ 1.011 0 Cluster B

1233 DGSDEQHCEPLCTHF 0.038 0 Cluster A

1237 EQHCEPLCTHFMDFV 0.388 0 Cluster A

1245 THFMDFVCKNRQQCL 0.339 0 Cluster A

1251 VCKNRQQCLFHSMVC 0.451 0 Cluster B

1258 CLFHSMVCDGIIQCR 1.228 0 Cluster B

1264 VCDGIIQCRDGSDED 2.109 0 Cluster B

1277 EDAAFAGCSQDPEFH 0.399 0 Cluster A

1287 DPEFHKVCDEFGFQC 0.641 0 Cluster B

1294 CDEFGFQCQNGVCIS 2.408 0 Cluster B

1299 FQCQNGVCISLIWKC 1.049 0 Cluster B

1306 CISLIWKCDGMDDCG 0.311 0 Cluster A

1312 KCDGMDDCGDYSDEA 0.94 0 Cluster B

1321 DYSDEANCENPTEAP 1.5 0 Cluster B

1330 NPTEAPNCSRYFQFR 0.257 0 Cluster A

1338 SRYFQFRCENGHCIP 0.202 0 Cluster A

1343 FRCENGHCIPNRWKC 0.429 0 Cluster B

1350 CIPNRWKCDRENDCG 0.598 0 Cluster B

1356 KCDRENDCGDWSDEK 0.766 0 Cluster B

1365 DWSDEKDCGDSHILP 0.929 0 Cluster B

1381 STPGPSTCLPNYYRC 0.717 0 Cluster B

1388 CLPNYYRCSSGTCVM 1.12 0 Cluster B

1393 YRCSSGTCVMDTWVC 15.212 0 Cluster C

1400 CVMDTWVCDGYRDCA 0.443 0 Cluster A

1406 VCDGYRDCADGSDEE 1.261 0 Cluster B

1415 DGSDEEACPLLANVT 22.956 0 Cluster C

1433 TPTQLGRCDRFEFEC 0.94 0 Cluster B

1440 CDRFEFECHQPKTCI 0.055 0 Cluster A

1446 ECHQPKTCIPNWKRC 0.603 0 Cluster B

1453 CIPNWKRCDGHQDCQ 0.457 0 Cluster B

1459 RCDGHQDCQDGRDEA 0.186 0 Cluster A

1468 DGRDEANCPTHSTLT 0.219 0 Cluster A

1476 PTHSTLTCMSREFQC 2.174 0 Cluster B

1483 CMSREFQCEDGEACI 0.957 0 Cluster B

1489 QCEDGEACIVLSERC 20.015 0 Cluster C

1496 CIVLSERCDGFLDCS 0.175 0 Cluster A

1502 RCDGFLDCSDESDEK 2.766 0 Cluster B

1511 DESDEKACSDELTVY 0.246 0 Cluster A

1548 KKMPSASCVYNVYYR 1.679 0 Cluster B

1593 QVKVQVQCLSKAHNT 2.989 0 Cluster B

1822 INQTAVECTWTGPRN 1.136 0 Cluster B

2063 TFTVQARCLFGNQIC 0.885 0 Cluster A

2070 CLFGNQICGEPAILL 2.174 0 Cluster B

**SORL1 Phosphorylation Data**

>Sequence 2220 amino acids

# **netphos-3.1b prediction results**

# Sequence # x Context Score Kinase Answer

-------------------------------------------------------------------

1. # Sequence 15 T RELATEDRE 0.635 CKII YES
2. # Sequence 15 T RELATEDRE 0.518 unsp YES
3. # Sequence 23 T ECEPTXRIS 0.653 unsp YES
4. # Sequence 27 S TXRISXFXR 0.743 PKA YES
5. # Sequence 27 S TXRISXFXR 0.558 unsp YES
6. # Sequence 44 S PIENSMATR 0.558 DNAPK YES
7. # Sequence 49 S MATRSSRRE 0.958 unsp YES
8. # Sequence 49 S MATRSSRRE 0.824 PKC YES
9. # Sequence 50 S ATRSSRRES 0.998 unsp YES
10. # Sequence 50 S ATRSSRRES 0.670 PKC YES
11. # Sequence 54 S SRRESRLPF 0.994 unsp YES
12. # Sequence 54 S SRRESRLPF 0.853 PKA YES
13. # Sequence 54 S SRRESRLPF 0.618 PKG YES
14. # Sequence 114 S ARGASRADE 0.996 unsp YES
15. # Sequence 114 S ARGASRADE 0.699 PKA YES
16. # Sequence 126 S RRKRSAALQ 0.861 PKA YES
17. # Sequence 126 S RRKRSAALQ 0.720 PKG YES
18. # Sequence 126 S RRKRSAALQ 0.560 unsp YES
19. # Sequence 126 S RRKRSAALQ 0.554 RSK YES
20. # Sequence 141 S YGQVSLNDS 0.907 unsp YES
21. # Sequence 168 S LARDSLALA 0.664 PKA YES
22. # Sequence 168 S LARDSLALA 0.553 unsp YES
23. # Sequence 176 S ARPKSSDVY 0.810 unsp YES
24. # Sequence 176 S ARPKSSDVY 0.562 PKA YES
25. # Sequence 176 S ARPKSSDVY 0.501 PKG YES
26. # Sequence 180 Y SSDVYVSYD 0.986 unsp YES
27. # Sequence 180 Y SSDVYVSYD 0.586 INSR YES
28. # Sequence 182 S DVYVSYDYG 0.797 unsp YES
29. # Sequence 183 Y VYVSYDYGK 0.505 INSR YES
30. # Sequence 188 S DYGKSFKKI 0.899 PKC YES
31. # Sequence 193 S FKKISDKLN 0.931 unsp YES
32. # Sequence 193 S FKKISDKLN 0.573 PKA YES
33. # Sequence 214 S QFYHSPADN 0.997 unsp YES
34. # Sequence 221 Y DNKRYIFAD 0.655 unsp YES
35. # Sequence 227 Y FADAYAQYL 0.886 unsp YES
36. # Sequence 227 Y FADAYAQYL 0.505 INSR YES
37. # Sequence 245 S LQGFSIPFR 0.503 cdc2 YES
38. # Sequence 260 S HSKASNLLL 0.637 PKA YES
39. # Sequence 284 T DFGQTWIMI 0.629 PKC YES
40. # Sequence 296 S VKSFSWGID 0.529 CKI YES
41. # Sequence 302 Y GIDPYDKPN 0.519 INSR YES
42. # Sequence 302 Y GIDPYDKPN 0.502 unsp YES
43. # Sequence 307 T DKPNTIYIE 0.652 unsp YES
44. # Sequence 309 Y PNTIYIERH 0.823 unsp YES
45. # Sequence 309 Y PNTIYIERH 0.607 EGFR YES
46. # Sequence 316 S RHEPSGYST 0.954 unsp YES
47. # Sequence 318 Y EPSGYSTVF 0.585 EGFR YES
48. # Sequence 318 Y EPSGYSTVF 0.537 unsp YES
49. # Sequence 319 S PSGYSTVFR 0.547 cdc2 YES
50. # Sequence 320 T SGYSTVFRS 0.830 PKC YES
51. # Sequence 325 T VFRSTDFFQ 0.536 PKC YES
52. # Sequence 330 S DFFQSRENQ 0.987 unsp YES
53. # Sequence 330 S DFFQSRENQ 0.513 CKII YES
54. # Sequence 350 Y LRDKYMFAT 0.533 unsp YES
55. # Sequence 354 T YMFATKVVH 0.644 PKC YES
56. # Sequence 354 T YMFATKVVH 0.518 unsp YES
57. # Sequence 362 S HLLGSEQQS 0.649 PKC YES
58. # Sequence 373 S QLWVSFGRK 0.794 PKC YES
59. # Sequence 394 Y INEYYIADA 0.737 unsp YES
60. # Sequence 417 Y RTNLYISEA 0.724 unsp YES
61. # Sequence 417 Y RTNLYISEA 0.554 INSR YES
62. # Sequence 419 S NLYISEAEG 0.980 unsp YES
63. # Sequence 419 S NLYISEAEG 0.617 CKII YES
64. # Sequence 427 S GLKFSLSLE 0.691 PKA YES
65. # Sequence 427 S GLKFSLSLE 0.550 cdc2 YES
66. # Sequence 429 S KFSLSLENV 0.536 PKA YES
67. # Sequence 429 S KFSLSLENV 0.524 unsp YES
68. # Sequence 435 Y ENVLYYSPG 0.920 unsp YES
69. # Sequence 436 Y NVLYYSPGG 0.511 INSR YES
70. # Sequence 437 S VLYYSPGGA 0.555 CKI YES
71. # Sequence 437 S VLYYSPGGA 0.539 cdk5 YES
72. # Sequence 437 S VLYYSPGGA 0.520 GSK3 YES
73. # Sequence 437 S VLYYSPGGA 0.516 cdc2 YES
74. # Sequence 445 T AGSDTLVRY 0.575 cdc2 YES
75. # Sequence 476 S LINGSMNEE 0.643 CKII YES
76. # Sequence 476 S LINGSMNEE 0.586 DNAPK YES
77. # Sequence 487 T RSVITFDKG 0.690 PKC YES
78. # Sequence 505 Y AFTGYGEKI 0.526 EGFR YES
79. # Sequence 514 S NCELSQGCS 0.797 unsp YES
80. # Sequence 514 S NCELSQGCS 0.605 DNAPK YES
81. # Sequence 514 S NCELSQGCS 0.527 ATM YES
82. # Sequence 526 S AQRLSQLLN 0.652 PKA YES
83. # Sequence 526 S AQRLSQLLN 0.599 ATM YES
84. # Sequence 526 S AQRLSQLLN 0.571 cdc2 YES
85. # Sequence 526 S AQRLSQLLN 0.564 DNAPK YES
86. # Sequence 540 S MPILSKESA 0.973 unsp YES
87. # Sequence 551 T LIIATGSVG 0.515 cdc2 YES
88. # Sequence 553 S IATGSVGKN 0.899 PKC YES
89. # Sequence 560 S KNLASKTNV 0.560 cdc2 YES
90. # Sequence 565 Y KTNVYISSS 0.511 unsp YES
91. # Sequence 567 S NVYISSSAG 0.952 unsp YES
92. # Sequence 569 S YISSSAGAR 0.561 PKC YES
93. # Sequence 583 Y PGPHYYTWG 0.555 unsp YES
94. # Sequence 607 Y NELKYSTNE 0.928 unsp YES
95. # Sequence 608 S ELKYSTNEG 0.932 unsp YES
96. # Sequence 608 S ELKYSTNEG 0.613 CKII YES
97. # Sequence 609 T LKYSTNEGE 0.504 CKII YES
98. # Sequence 614 T NEGETWKTF 0.598 unsp YES
99. # Sequence 621 S TFIFSEKPV 0.632 unsp YES
100. # Sequence 621 S TFIFSEKPV 0.565 PKC YES
101. # Sequence 621 S TFIFSEKPV 0.502 PKG YES
102. # Sequence 642 T STVFTIFGS 0.574 CKI YES
103. # Sequence 642 T STVFTIFGS 0.502 PKC YES
104. # Sequence 646 S TIFGSNKEN 0.916 unsp YES
105. # Sequence 646 S TIFGSNKEN 0.723 PKC YES
106. # Sequence 646 S TIFGSNKEN 0.555 cdc2 YES
107. # Sequence 653 S ENVHSWLIL 0.506 PKA YES
108. # Sequence 674 Y TENDYKLWS 0.805 unsp YES
109. # Sequence 678 S YKLWSPSDE 0.997 unsp YES
110. # Sequence 680 S LWSPSDERG 0.920 unsp YES
111. # Sequence 680 S LWSPSDERG 0.505 CKII YES
112. # Sequence 699 T FKRRTPHAT 0.937 unsp YES
113. # Sequence 699 T FKRRTPHAT 0.529 p38MAPK YES
114. # Sequence 726 Y TREDYECDF 0.655 unsp YES
115. # Sequence 726 Y TREDYECDF 0.532 INSR YES
116. # Sequence 735 S GFKMSEDLS 0.625 CKII YES
117. # Sequence 739 S SEDLSLEVC 0.910 unsp YES
118. # Sequence 739 S SEDLSLEVC 0.531 DNAPK YES
119. # Sequence 750 S DPEFSGKSY 0.957 unsp YES
120. # Sequence 753 S FSGKSYSPP 0.882 unsp YES
121. # Sequence 755 S GKSYSPPVP 0.706 unsp YES
122. # Sequence 755 S GKSYSPPVP 0.550 cdk5 YES
123. # Sequence 755 S GKSYSPPVP 0.540 p38MAPK YES
124. # Sequence 765 T PVGSTYRRT 0.925 unsp YES
125. # Sequence 765 T PVGSTYRRT 0.870 PKC YES
126. # Sequence 769 T TYRRTRGYR 0.752 PKC YES
127. # Sequence 776 S YRKISGDTC 0.705 PKA YES
128. # Sequence 776 S YRKISGDTC 0.649 unsp YES
129. # Sequence 776 S YRKISGDTC 0.530 cdc2 YES
130. # Sequence 781 S GDTCSGGDV 0.777 unsp YES
131. # Sequence 812 S AVRKSIYRY 0.995 unsp YES
132. # Sequence 812 S AVRKSIYRY 0.764 PKA YES
133. # Sequence 841 Y LDFDYEHNC 0.777 unsp YES
134. # Sequence 865 T LNGSTGQEV 0.515 CKII YES
135. # Sequence 873 S VIINSGLET 0.607 CKII YES
136. # Sequence 887 S FEPLSQLLY 0.538 ATM YES
137. # Sequence 887 S FEPLSQLLY 0.513 DNAPK YES
138. # Sequence 912 T DFRLTIVNS 0.921 unsp YES
139. # Sequence 917 S IVNSSVLDR 0.984 unsp YES
140. # Sequence 917 S IVNSSVLDR 0.685 PKC YES
141. # Sequence 937 T VMFWTDWGD 0.526 CKII YES
142. # Sequence 949 S GIYRSNMDG 0.823 unsp YES
143. # Sequence 978 Y DQWIYWTDA 0.503 INSR YES
144. # Sequence 980 T WIYWTDAYL 0.556 CKII YES
145. # Sequence 993 S RITFSGQQR 0.759 PKC YES
146. # Sequence 1018 Y KNEIYWDDW 0.929 unsp YES
147. # Sequence 1023 S WDDWSQLSI 0.597 DNAPK YES
148. # Sequence 1023 S WDDWSQLSI 0.535 ATM YES
149. # Sequence 1026 S WSQLSIFRA 0.509 CKI YES
150. # Sequence 1031 S IFRASKYSG 0.992 unsp YES
151. # Sequence 1031 S IFRASKYSG 0.589 PKA YES
152. # Sequence 1031 S IFRASKYSG 0.587 PKC YES
153. # Sequence 1034 S ASKYSGSQM 0.951 unsp YES
154. # Sequence 1036 S KYSGSQMEI 0.705 unsp YES
155. # Sequence 1036 S KYSGSQMEI 0.628 DNAPK YES
156. # Sequence 1036 S KYSGSQMEI 0.527 cdc2 YES
157. # Sequence 1060 T KGKNTGSNA 0.559 PKA YES
158. # Sequence 1071 S PRPCSLLCL 0.741 PKA YES
159. # Sequence 1081 S KANNSRSCR 0.526 PKA YES
160. # Sequence 1083 S NNSRSCRCP 0.946 unsp YES
161. # Sequence 1092 S EDVSSSVLP 0.509 cdc2 YES
162. # Sequence 1093 S DVSSSVLPS 0.533 PKC YES
163. # Sequence 1093 S DVSSSVLPS 0.512 cdc2 YES
164. # Sequence 1121 T KQATTICDL 0.897 unsp YES
165. # Sequence 1134 S RCQESGTCI 0.561 unsp YES
166. # Sequence 1134 S RCQESGTCI 0.506 CaM-II YES
167. # Sequence 1141 S CIPLSYKCD 0.987 unsp YES
168. # Sequence 1141 S CIPLSYKCD 0.837 PKC YES
169. # Sequence 1154 S CGDNSDESH 0.971 unsp YES
170. # Sequence 1154 S CGDNSDESH 0.664 CKII YES
171. # Sequence 1157 S NSDESHCEM 0.994 unsp YES
172. # Sequence 1157 S NSDESHCEM 0.593 CKII YES
173. # Sequence 1166 S HQCRSDEYN 0.621 unsp YES
174. # Sequence 1166 S HQCRSDEYN 0.510 PKC YES
175. # Sequence 1169 Y RSDEYNCSS 0.915 unsp YES
176. # Sequence 1169 Y RSDEYNCSS 0.517 SRC YES
177. # Sequence 1172 S EYNCSSGMC 0.532 cdc2 YES
178. # Sequence 1173 S YNCSSGMCI 0.551 cdc2 YES
179. # Sequence 1180 S CIRSSWVCD 0.961 unsp YES
180. # Sequence 1180 S CIRSSWVCD 0.669 PKC YES
181. # Sequence 1180 S CIRSSWVCD 0.567 PKA YES
182. # Sequence 1193 S CRDWSDEAN 0.987 unsp YES
183. # Sequence 1193 S CRDWSDEAN 0.598 PKA YES
184. # Sequence 1199 T EANCTAIYH 0.507 PKC YES
185. # Sequence 1228 T CDGDTDCQD 0.512 CKII YES
186. # Sequence 1234 S CQDGSDEDP 0.996 unsp YES
187. # Sequence 1234 S CQDGSDEDP 0.577 CKII YES
188. # Sequence 1234 S CQDGSDEDP 0.517 ATM YES
189. # Sequence 1254 T CPNGTCIPS 0.524 PKC YES
190. # Sequence 1259 S CIPSSKHCD 0.779 unsp YES
191. # Sequence 1259 S CIPSSKHCD 0.698 PKC YES
192. # Sequence 1269 S LRDCSDGSD 0.946 unsp YES
193. # Sequence 1269 S LRDCSDGSD 0.520 PKA YES
194. # Sequence 1272 S CSDGSDEQH 0.978 unsp YES
195. # Sequence 1272 S CSDGSDEQH 0.604 CKII YES
196. # Sequence 1312 S CRDGSDEDA 0.997 unsp YES
197. # Sequence 1312 S CRDGSDEDA 0.508 RSK YES
198. # Sequence 1322 S FAGCSQDPE 0.531 DNAPK YES
199. # Sequence 1322 S FAGCSQDPE 0.516 ATM YES
200. # Sequence 1345 S GVCISLIWK 0.560 PKC YES
201. # Sequence 1359 Y DCGDYSDEA 0.967 unsp YES
202. # Sequence 1360 S CGDYSDEAN 0.922 unsp YES
203. # Sequence 1360 S CGDYSDEAN 0.682 CKII YES
204. # Sequence 1404 S CGDWSDEKD 0.847 unsp YES
205. # Sequence 1404 S CGDWSDEKD 0.618 CKII YES
206. # Sequence 1418 S ILPFSTPGP 0.546 cdc2 YES
207. # Sequence 1418 S ILPFSTPGP 0.502 GSK3 YES
208. # Sequence 1419 T LPFSTPGPS 0.821 unsp YES
209. # Sequence 1433 S YYRCSSGTC 0.618 PKA YES
210. # Sequence 1434 S YRCSSGTCV 0.714 PKA YES
211. # Sequence 1434 S YRCSSGTCV 0.526 RSK YES
212. # Sequence 1447 Y VCDGYRDCA 0.875 unsp YES
213. # Sequence 1454 S CADGSDEEA 0.993 unsp YES
214. # Sequence 1454 S CADGSDEEA 0.699 CKII YES
215. # Sequence 1454 S CADGSDEEA 0.519 cdc2 YES
216. # Sequence 1470 T TAASTPTQL 0.651 cdk5 YES
217. # Sequence 1470 T TAASTPTQL 0.585 unsp YES
218. # Sequence 1470 T TAASTPTQL 0.536 p38MAPK YES
219. # Sequence 1489 T HQPKTCIPN 0.737 PKC YES
220. # Sequence 1519 T HSTLTCMSR 0.831 PKC YES
221. # Sequence 1522 S LTCMSREFQ 0.945 unsp YES
222. # Sequence 1522 S LTCMSREFQ 0.531 CKI YES
223. # Sequence 1537 S CIVLSERCD 0.699 PKC YES
224. # Sequence 1547 S FLDCSDESD 0.723 unsp YES
225. # Sequence 1547 S FLDCSDESD 0.655 CKII YES
226. # Sequence 1547 S FLDCSDESD 0.520 cdc2 YES
227. # Sequence 1550 S CSDESDEKA 0.989 unsp YES
228. # Sequence 1550 S CSDESDEKA 0.588 CKII YES
229. # Sequence 1556 S EKACSDELT 0.968 unsp YES
230. # Sequence 1556 S EKACSDELT 0.575 CKII YES
231. # Sequence 1560 T SDELTVYKV 0.723 unsp YES
232. # Sequence 1560 T SDELTVYKV 0.718 PKC YES
233. # Sequence 1580 T DVTLTWMRP 0.824 PKC YES
234. # Sequence 1589 S KKMPSASCV 0.675 PKA YES
235. # Sequence 1589 S KKMPSASCV 0.562 PKG YES
236. # Sequence 1598 Y YNVYYRVVG 0.538 unsp YES
237. # Sequence 1604 S VVGESIWKT 0.551 unsp YES
238. # Sequence 1611 T KTLETHSNK 0.811 PKC YES
239. # Sequence 1613 S LETHSNKTN 0.963 unsp YES
240. # Sequence 1616 T HSNKTNTVL 0.643 PKC YES
241. # Sequence 1618 T NKTNTVLKV 0.722 PKC YES
242. # Sequence 1627 T LKPDTTYQV 0.554 unsp YES
243. # Sequence 1629 Y PDTTYQVKV 0.628 unsp YES
244. # Sequence 1629 Y PDTTYQVKV 0.513 SRC YES
245. # Sequence 1639 S VQCLSKAHN 0.750 PKC YES
246. # Sequence 1652 T VTLRTPEGL 0.638 p38MAPK YES
247. # Sequence 1652 T VTLRTPEGL 0.545 CKI YES
248. # Sequence 1652 T VTLRTPEGL 0.510 unsp YES
249. # Sequence 1652 T VTLRTPEGL 0.506 cdk5 YES
250. # Sequence 1666 S NLQLSLPRE 0.853 unsp YES
251. # Sequence 1696 Y YIVEYSRSG 0.508 INSR YES
252. # Sequence 1697 S IVEYSRSGS 0.588 unsp YES
253. # Sequence 1697 S IVEYSRSGS 0.546 CKI YES
254. # Sequence 1699 S EYSRSGSKM 0.543 cdc2 YES
255. # Sequence 1701 S SRSGSKMWA 0.977 unsp YES
256. # Sequence 1701 S SRSGSKMWA 0.577 PKC YES
257. # Sequence 1701 S SRSGSKMWA 0.524 PKA YES
258. # Sequence 1701 S SRSGSKMWA 0.520 RSK YES
259. # Sequence 1706 S KMWASQRAA 0.832 PKC YES
260. # Sequence 1706 S KMWASQRAA 0.636 DNAPK YES
261. # Sequence 1706 S KMWASQRAA 0.538 unsp YES
262. # Sequence 1711 S QRAASNFTE 0.861 unsp YES
263. # Sequence 1714 T ASNFTEIKN 0.538 PKC YES
264. # Sequence 1726 T NTLYTVRVA 0.807 unsp YES
265. # Sequence 1726 T NTLYTVRVA 0.794 PKC YES
266. # Sequence 1733 T VAAVTSRGI 0.869 PKC YES
267. # Sequence 1733 T VAAVTSRGI 0.800 unsp YES
268. # Sequence 1734 S AAVTSRGIG 0.520 cdc2 YES
269. # Sequence 1741 S IGNWSDSKS 0.512 cdc2 YES
270. # Sequence 1743 S NWSDSKSIT 0.775 unsp YES
271. # Sequence 1743 S NWSDSKSIT 0.516 cdc2 YES
272. # Sequence 1747 T SKSITTIKG 0.783 PKC YES
273. # Sequence 1748 T KSITTIKGK 0.957 unsp YES
274. # Sequence 1748 T KSITTIKGK 0.842 PKC YES
275. # Sequence 1763 S IHIDSYGEN 0.968 unsp YES
276. # Sequence 1763 S IHIDSYGEN 0.621 CKII YES
277. # Sequence 1764 Y HIDSYGENY 0.638 unsp YES
278. # Sequence 1764 Y HIDSYGENY 0.530 INSR YES
279. # Sequence 1764 Y HIDSYGENY 0.505 EGFR YES
280. # Sequence 1768 Y YGENYLSFT 0.770 unsp YES
281. # Sequence 1784 Y KVNGYVVNL 0.686 unsp YES
282. # Sequence 1794 T WAFDTHKQE 0.960 unsp YES
283. # Sequence 1794 T WAFDTHKQE 0.635 PKC YES
284. # Sequence 1807 S NFRGSILSH 0.817 unsp YES
285. # Sequence 1807 S NFRGSILSH 0.728 PKC YES
286. # Sequence 1807 S NFRGSILSH 0.556 cdc2 YES
287. # Sequence 1810 S GSILSHKVG 0.808 PKC YES
288. # Sequence 1810 S GSILSHKVG 0.762 unsp YES
289. # Sequence 1822 Y AHTSYEISA 0.886 unsp YES
290. # Sequence 1835 S DLGDSPLAF 0.966 unsp YES
291. # Sequence 1853 S PPAPSLKAK 0.960 unsp YES
292. # Sequence 1853 S PPAPSLKAK 0.796 PKC YES
293. # Sequence 1862 T AINQTAVEC 0.506 CKII YES
294. # Sequence 1867 T AVECTWTGP 0.524 CKI YES
295. # Sequence 1876 Y RNVVYGIFY 0.519 EGFR YES
296. # Sequence 1882 T IFYATSFLD 0.508 CKII YES
297. # Sequence 1888 Y FLDLYRNPK 0.715 unsp YES
298. # Sequence 1893 S RNPKSLTTS 0.505 GSK3 YES
299. # Sequence 1895 T PKSLTTSLH 0.534 PKC YES
300. # Sequence 1896 T KSLTTSLHN 0.687 PKC YES
301. # Sequence 1897 S SLTTSLHNK 0.700 PKC YES
302. # Sequence 1897 S SLTTSLHNK 0.517 cdc2 YES
303. # Sequence 1902 T LHNKTVIVS 0.617 PKC YES
304. # Sequence 1906 S TVIVSKDEQ 0.971 unsp YES
305. # Sequence 1906 S TVIVSKDEQ 0.614 CKII YES
306. # Sequence 1906 S TVIVSKDEQ 0.571 PKG YES
307. # Sequence 1911 Y KDEQYLFLV 0.750 unsp YES
308. # Sequence 1928 Y PSSDYVVVK 0.972 unsp YES
309. # Sequence 1937 S MIPDSRLPP 0.610 unsp YES
310. # Sequence 1937 S MIPDSRLPP 0.506 GSK3 YES
311. # Sequence 1949 T HVVHTGKTS 0.827 PKC YES
312. # Sequence 1952 T HTGKTSVVI 0.759 PKC YES
313. # Sequence 1960 S IKWESPYDS 0.996 unsp YES
314. # Sequence 1960 S IKWESPYDS 0.546 p38MAPK YES
315. # Sequence 1960 S IKWESPYDS 0.526 GSK3 YES
316. # Sequence 1960 S IKWESPYDS 0.517 cdk5 YES
317. # Sequence 1962 Y WESPYDSPD 0.975 unsp YES
318. # Sequence 1964 S SPYDSPDQD 0.968 unsp YES
319. # Sequence 1964 S SPYDSPDQD 0.570 cdk5 YES
320. # Sequence 1964 S SPYDSPDQD 0.512 GSK3 YES
321. # Sequence 1964 S SPYDSPDQD 0.503 CKII YES
322. # Sequence 1964 S SPYDSPDQD 0.500 p38MAPK YES
323. # Sequence 1982 T LIRKTDRSY 0.846 PKC YES
324. # Sequence 1982 T LIRKTDRSY 0.695 unsp YES
325. # Sequence 1985 S KTDRSYKVK 0.993 unsp YES
326. # Sequence 1985 S KTDRSYKVK 0.906 PKC YES
327. # Sequence 1990 S YKVKSRNST 0.973 unsp YES
328. # Sequence 1993 S KSRNSTVEY 0.996 unsp YES
329. # Sequence 1993 S KSRNSTVEY 0.743 PKA YES
330. # Sequence 1997 Y STVEYTLNK 0.709 unsp YES
331. # Sequence 2008 Y PGGKYHIIV 0.544 unsp YES
332. # Sequence 2021 S MSKDSSIKI 0.703 PKC YES
333. # Sequence 2022 S SKDSSIKIT 0.994 unsp YES
334. # Sequence 2022 S SKDSSIKIT 0.812 PKC YES
335. # Sequence 2022 S SKDSSIKIT 0.518 PKA YES
336. # Sequence 2029 S ITTVSLSAP 0.695 unsp YES
337. # Sequence 2029 S ITTVSLSAP 0.621 PKA YES
338. # Sequence 2029 S ITTVSLSAP 0.561 PKC YES
339. # Sequence 2029 S ITTVSLSAP 0.511 cdc2 YES
340. # Sequence 2031 S TVSLSAPDA 0.993 unsp YES
341. # Sequence 2051 S LFWKSLALK 0.526 DNAPK YES
342. # Sequence 2062 S HFNESRGYE 0.516 PKA YES
343. # Sequence 2065 Y ESRGYEIHM 0.709 unsp YES
344. # Sequence 2072 S HMFDSAMNI 0.582 PKA YES
345. # Sequence 2091 S FFKISNLKM 0.505 PKG YES
346. # Sequence 2102 T NYTFTVQAR 0.816 PKC YES
347. # Sequence 2127 S DELGSGADA 0.915 unsp YES
348. # Sequence 2132 S GADASATQA 0.510 cdc2 YES
349. # Sequence 2134 T DASATQAAR 0.727 PKC YES
350. # Sequence 2134 T DASATQAAR 0.602 DNAPK YES
351. # Sequence 2139 S QAARSTDVA 0.958 unsp YES
352. # Sequence 2139 S QAARSTDVA 0.510 cdc2 YES
353. # Sequence 2140 T AARSTDVAA 0.657 unsp YES
354. # Sequence 2156 S LILLSLGVG 0.665 PKA YES
355. # Sequence 2166 T AILYTKHRR 0.782 PKC YES
356. # Sequence 2173 S RRLQSSFTA 0.612 PKA YES
357. # Sequence 2173 S RRLQSSFTA 0.513 RSK YES
358. # Sequence 2176 T QSSFTAFAN 0.664 PKC YES
359. # Sequence 2176 T QSSFTAFAN 0.523 cdc2 YES
360. # Sequence 2181 S AFANSHYSS 0.981 unsp YES
361. # Sequence 2183 Y ANSHYSSRL 0.523 unsp YES
362. # Sequence 2189 S SRLGSAIFS 0.942 unsp YES
363. # Sequence 2189 S SRLGSAIFS 0.625 PKC YES
364. # Sequence 2189 S SRLGSAIFS 0.595 RSK YES
365. # Sequence 2193 S SAIFSSGDD 0.968 unsp YES
366. # Sequence 2193 S SAIFSSGDD 0.542 CKI YES
367. # Sequence 2194 S AIFSSGDDL 0.772 unsp YES
368. # Sequence 2194 S AIFSSGDDL 0.503 CKII YES


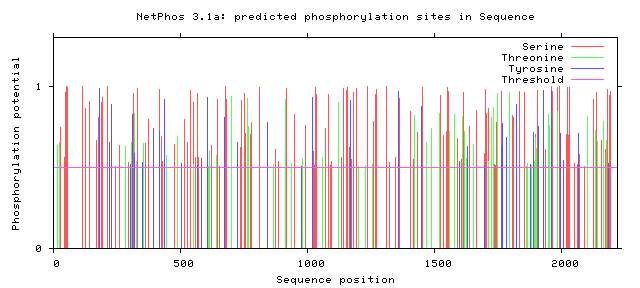


**SORL1 S-Nitrosylation Data**

**SeqName Position Potential Jury N-Glyc**

**agreement result**

**----------------------------------------------------------------------**

1. **XP_011541265.1 99 NDSH 0.5012 (3/9) +**
2. **XP_011541265.1 158 NRSE 0.7037 (8/9) +**
3. **XP_011541265.1 368 NRTN 0.5074 (4/9) +**
4. **XP_011541265.1 430 NGSM 0.5284 (7/9) +**
5. **XP_011541265.1 616 NATD 0.5957 (7/9) +**
6. **XP_011541265.1 674 NCSC 0.5378 (4/9) +**
7. **XP_011541265.1 818 NGST 0.5867 (8/9) +**
8. **XP_011541265.1 871 NSSV 0.5939 (6/9) +**
9. **XP_011541265.1 1035 NNSR 0.3798 (7/9) -**
10. **XP_011541265.1 1068 NNTC 0.5431 (5/9) +**
11. **XP_011541265.1 1126 NCSS 0.4689 (4/9) -**
12. **XP_011541265.1 1153 NCTA 0.4519 (5/9) -**
13. **XP_011541265.1 1208 NGTC 0.7360 (9/9) ++**
14. **XP_011541265.1 1323 NPTE 0.5529 (6/9) + WARNING: PRO-X1.**
15. **XP_011541265.1 1329 NCSR 0.6137 (8/9) +**
16. **XP_011541265.1 1420 NVTA 0.6751 (9/9) ++**
17. **XP_011541265.1 1570 NKTN 0.6137 (9/9) ++**
18. **XP_011541265.1 1668 NFTE 0.6132 (7/9) +**
19. **XP_011541265.1 1695 NWSD 0.5100 (4/9) +**
20. **XP_011541265.1 1771 NLTA 0.5702 (8/9) +**
21. **XP_011541265.1 1816 NQTA 0.5386 (5/9) +**
22. **XP_011541265.1 1856 NKTV 0.7157 (9/9) ++**
23. **XP_011541265.1 1948 NSTV 0.4332 (6/9) -**
24. **XP_011541265.1 1972 NMSK 0.4461 (5/9) -**
25. **XP_011541265.1 2016 NESR 0.3720 (8/9) -**
26. **XP_011541265.1 2031 NITA 0.6076 (8/9) +**
27. **XP_011541265.1 2038 NTTD 0.5536 (6/9) +**
28. **XP_011541265.1 2054 NYTF 0.5618 (7/9) +**


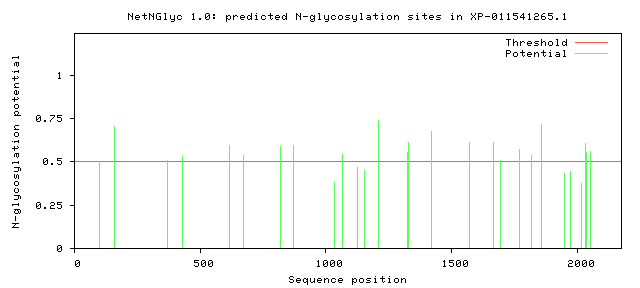


**SORL1 Glycosylation Data**

##gff-version 2

##source-version NetOGlyc 4.0.0.13

##date 18-9-7

##Type Protein

#seqname source feature start end score strand frame comment

1. XP_011541265_1 netOGlyc-4.0.0.13 CARBOHYD 3 3 0.80725 . . #POSITIVE
2. XP_011541265_1 netOGlyc-4.0.0.13 CARBOHYD 5 5 0.602693 . . #POSITIVE
3. XP_011541265_1 netOGlyc-4.0.0.13 CARBOHYD 6 6 0.785268 . . #POSITIVE
4. XP_011541265_1 netOGlyc-4.0.0.13 CARBOHYD 10 10 0.841581 . . #POSITIVE
5. XP_011541265_1 netOGlyc-4.0.0.13 CARBOHYD 17 17 0.15978 . .
6. XP_011541265_1 netOGlyc-4.0.0.13 CARBOHYD 32 32 0.229482 . .
7. XP_011541265_1 netOGlyc-4.0.0.13 CARBOHYD 39 39 0.686025 . . #POSITIVE
8. XP_011541265_1 netOGlyc-4.0.0.13 CARBOHYD 70 70 0.952384 . . #POSITIVE
9. XP_011541265_1 netOGlyc-4.0.0.13 CARBOHYD 82 82 0.835802 . . #POSITIVE
10. XP_011541265_1 netOGlyc-4.0.0.13 CARBOHYD 97 97 0.124927 . .
11. XP_011541265_1 netOGlyc-4.0.0.13 CARBOHYD 101 101 0.0528375 . .
12. XP_011541265_1 netOGlyc-4.0.0.13 CARBOHYD 114 114 0.00750324 . .
13. XP_011541265_1 netOGlyc-4.0.0.13 CARBOHYD 124 124 0.0665717 . .
14. XP_011541265_1 netOGlyc-4.0.0.13 CARBOHYD 132 132 0.0346788 . .
15. XP_011541265_1 netOGlyc-4.0.0.13 CARBOHYD 133 133 0.120659 . .
16. XP_011541265_1 netOGlyc-4.0.0.13 CARBOHYD 138 138 0.158144 . .
17. XP_011541265_1 netOGlyc-4.0.0.13 CARBOHYD 144 144 0.0658795 . .
18. XP_011541265_1 netOGlyc-4.0.0.13 CARBOHYD 149 149 0.0633106 . .
19. XP_011541265_1 netOGlyc-4.0.0.13 CARBOHYD 160 160 0.143563 . .
20. XP_011541265_1 netOGlyc-4.0.0.13 CARBOHYD 170 170 0.081485 . .
21. XP_011541265_1 netOGlyc-4.0.0.13 CARBOHYD 190 190 0.0164657 . .
22. XP_011541265_1 netOGlyc-4.0.0.13 CARBOHYD 196 196 0.0220769 . .
23. XP_011541265_1 netOGlyc-4.0.0.13 CARBOHYD 201 201 0.072645 . .
24. XP_011541265_1 netOGlyc-4.0.0.13 CARBOHYD 213 213 0.0757298 . .
25. XP_011541265_1 netOGlyc-4.0.0.13 CARBOHYD 216 216 0.0474926 . .
26. XP_011541265_1 netOGlyc-4.0.0.13 CARBOHYD 225 225 0.0393634 . .
27. XP_011541265_1 netOGlyc-4.0.0.13 CARBOHYD 234 234 0.0349354 . .
28. XP_011541265_1 netOGlyc-4.0.0.13 CARBOHYD 240 240 0.0248776 . .
29. XP_011541265_1 netOGlyc-4.0.0.13 CARBOHYD 250 250 0.0370572 . .
30. XP_011541265_1 netOGlyc-4.0.0.13 CARBOHYD 252 252 0.0942586 . .
31. XP_011541265_1 netOGlyc-4.0.0.13 CARBOHYD 263 263 0.0259762 . .
32. XP_011541265_1 netOGlyc-4.0.0.13 CARBOHYD 272 272 0.162587 . .
33. XP_011541265_1 netOGlyc-4.0.0.13 CARBOHYD 275 275 0.0639563 . .
34. XP_011541265_1 netOGlyc-4.0.0.13 CARBOHYD 276 276 0.0475624 . .
35. XP_011541265_1 netOGlyc-4.0.0.13 CARBOHYD 280 280 0.112625 . .
36. XP_011541265_1 netOGlyc-4.0.0.13 CARBOHYD 281 281 0.0596886 . .
37. XP_011541265_1 netOGlyc-4.0.0.13 CARBOHYD 286 286 0.122327 . .
38. XP_011541265_1 netOGlyc-4.0.0.13 CARBOHYD 310 310 0.063921 . .
39. XP_011541265_1 netOGlyc-4.0.0.13 CARBOHYD 318 318 0.0620823 . .
40. XP_011541265_1 netOGlyc-4.0.0.13 CARBOHYD 322 322 0.0440517 . .
41. XP_011541265_1 netOGlyc-4.0.0.13 CARBOHYD 323 323 0.0335654 . .
42. XP_011541265_1 netOGlyc-4.0.0.13 CARBOHYD 329 329 0.202552 . .
43. XP_011541265_1 netOGlyc-4.0.0.13 CARBOHYD 342 342 0.374453 . .
44. XP_011541265_1 netOGlyc-4.0.0.13 CARBOHYD 355 355 0.00615633 . .
45. XP_011541265_1 netOGlyc-4.0.0.13 CARBOHYD 364 364 0.0464252 . .
46. XP_011541265_1 netOGlyc-4.0.0.13 CARBOHYD 366 366 0.00814828 . .
47. XP_011541265_1 netOGlyc-4.0.0.13 CARBOHYD 370 370 0.00921145 . .
48. XP_011541265_1 netOGlyc-4.0.0.13 CARBOHYD 375 375 0.0171566 . .
49. XP_011541265_1 netOGlyc-4.0.0.13 CARBOHYD 383 383 0.0278143 . .
50. XP_011541265_1 netOGlyc-4.0.0.13 CARBOHYD 385 385 0.0293422 . .
51. XP_011541265_1 netOGlyc-4.0.0.13 CARBOHYD 393 393 0.347238 . .
52. XP_011541265_1 netOGlyc-4.0.0.13 CARBOHYD 399 399 0.126058 . .
53. XP_011541265_1 netOGlyc-4.0.0.13 CARBOHYD 401 401 0.0889892 . .
54. XP_011541265_1 netOGlyc-4.0.0.13 CARBOHYD 427 427 0.00595576 . .
55. XP_011541265_1 netOGlyc-4.0.0.13 CARBOHYD 432 432 0.00994577 . .
56. XP_011541265_1 netOGlyc-4.0.0.13 CARBOHYD 440 440 0.0600709 . .
57. XP_011541265_1 netOGlyc-4.0.0.13 CARBOHYD 443 443 0.0252196 . .
58. XP_011541265_1 netOGlyc-4.0.0.13 CARBOHYD 449 449 0.144229 . .
59. XP_011541265_1 netOGlyc-4.0.0.13 CARBOHYD 459 459 0.291398 . .
60. XP_011541265_1 netOGlyc-4.0.0.13 CARBOHYD 470 470 0.0405724 . .
61. XP_011541265_1 netOGlyc-4.0.0.13 CARBOHYD 474 474 0.069505 . .
62. XP_011541265_1 netOGlyc-4.0.0.13 CARBOHYD 482 482 0.0766197 . .
63. XP_011541265_1 netOGlyc-4.0.0.13 CARBOHYD 496 496 0.115168 . .
64. XP_011541265_1 netOGlyc-4.0.0.13 CARBOHYD 499 499 0.176847 . .
65. XP_011541265_1 netOGlyc-4.0.0.13 CARBOHYD 507 507 0.0943673 . .
66. XP_011541265_1 netOGlyc-4.0.0.13 CARBOHYD 509 509 0.0771683 . .
67. XP_011541265_1 netOGlyc-4.0.0.13 CARBOHYD 516 516 0.0657526 . .
68. XP_011541265_1 netOGlyc-4.0.0.13 CARBOHYD 518 518 0.0207215 . .
69. XP_011541265_1 netOGlyc-4.0.0.13 CARBOHYD 523 523 0.223685 . .
70. XP_011541265_1 netOGlyc-4.0.0.13 CARBOHYD 524 524 0.0886917 . .
71. XP_011541265_1 netOGlyc-4.0.0.13 CARBOHYD 525 525 0.0555732 . .
72. XP_011541265_1 netOGlyc-4.0.0.13 CARBOHYD 541 541 0.0931351 . .
73. XP_011541265_1 netOGlyc-4.0.0.13 CARBOHYD 550 550 0.0124056 . .
74. XP_011541265_1 netOGlyc-4.0.0.13 CARBOHYD 558 558 0.0251882 . .
75. XP_011541265_1 netOGlyc-4.0.0.13 CARBOHYD 564 564 0.141081 . .
76. XP_011541265_1 netOGlyc-4.0.0.13 CARBOHYD 565 565 0.109833 . .
77. XP_011541265_1 netOGlyc-4.0.0.13 CARBOHYD 570 570 0.0249243 . .
78. XP_011541265_1 netOGlyc-4.0.0.13 CARBOHYD 573 573 0.0133093 . .
79. XP_011541265_1 netOGlyc-4.0.0.13 CARBOHYD 577 577 0.0171055 . .
80. XP_011541265_1 netOGlyc-4.0.0.13 CARBOHYD 588 588 0.00885248 . .
81. XP_011541265_1 netOGlyc-4.0.0.13 CARBOHYD 594 594 0.101604 . .
82. XP_011541265_1 netOGlyc-4.0.0.13 CARBOHYD 595 595 0.0397353 . .
83. XP_011541265_1 netOGlyc-4.0.0.13 CARBOHYD 598 598 0.0158831 . .
84. XP_011541265_1 netOGlyc-4.0.0.13 CARBOHYD 602 602 0.0310149 . .
85. XP_011541265_1 netOGlyc-4.0.0.13 CARBOHYD 609 609 0.0160047 . .
86. XP_011541265_1 netOGlyc-4.0.0.13 CARBOHYD 618 618 0.305577 . .
87. XP_011541265_1 netOGlyc-4.0.0.13 CARBOHYD 626 626 0.512808 . . #POSITIVE
88. XP_011541265_1 netOGlyc-4.0.0.13 CARBOHYD 634 634 0.478832 . .
89. XP_011541265_1 netOGlyc-4.0.0.13 CARBOHYD 636 636 0.421836 . .
90. XP_011541265_1 netOGlyc-4.0.0.13 CARBOHYD 649 649 0.429576 . .
91. XP_011541265_1 netOGlyc-4.0.0.13 CARBOHYD 655 655 0.853181 . . #POSITIVE
92. XP_011541265_1 netOGlyc-4.0.0.13 CARBOHYD 659 659 0.837149 . . #POSITIVE
93. XP_011541265_1 netOGlyc-4.0.0.13 CARBOHYD 673 673 0.489693 . .
94. XP_011541265_1 netOGlyc-4.0.0.13 CARBOHYD 676 676 0.249907 . .
95. XP_011541265_1 netOGlyc-4.0.0.13 CARBOHYD 678 678 0.286974 . .
96. XP_011541265_1 netOGlyc-4.0.0.13 CARBOHYD 691 691 0.110767 . .
97. XP_011541265_1 netOGlyc-4.0.0.13 CARBOHYD 695 695 0.260215 . .
98. XP_011541265_1 netOGlyc-4.0.0.13 CARBOHYD 706 706 0.807255 . . #POSITIVE
99. XP_011541265_1 netOGlyc-4.0.0.13 CARBOHYD 709 709 0.88949 . . #POSITIVE
100. XP_011541265_1 netOGlyc-4.0.0.13 CARBOHYD 711 711 0.75787 . . #POSITIVE
101. XP_011541265_1 netOGlyc-4.0.0.13 CARBOHYD 720 720 0.854586 . . #POSITIVE
102. XP_011541265_1 netOGlyc-4.0.0.13 CARBOHYD 721 721 0.720637 . . #POSITIVE
103. XP_011541265_1 netOGlyc-4.0.0.13 CARBOHYD 725 725 0.531224 . . #POSITIVE
104. XP_011541265_1 netOGlyc-4.0.0.13 CARBOHYD 732 732 0.580803 . . #POSITIVE
105. XP_011541265_1 netOGlyc-4.0.0.13 CARBOHYD 735 735 0.417101 . .
106. XP_011541265_1 netOGlyc-4.0.0.13 CARBOHYD 737 737 0.285688 . .
107. XP_011541265_1 netOGlyc-4.0.0.13 CARBOHYD 768 768 0.0588602 . .
108. XP_011541265_1 netOGlyc-4.0.0.13 CARBOHYD 776 776 0.0669588 . .
109. XP_011541265_1 netOGlyc-4.0.0.13 CARBOHYD 779 779 0.0282817 . .
110. XP_011541265_1 netOGlyc-4.0.0.13 CARBOHYD 785 785 0.032691 . .
111. XP_011541265_1 netOGlyc-4.0.0.13 CARBOHYD 805 805 0.00491662 . .
112. XP_011541265_1 netOGlyc-4.0.0.13 CARBOHYD 820 820 0.037979 . .
113. XP_011541265_1 netOGlyc-4.0.0.13 CARBOHYD 821 821 0.0148475 . .
114. XP_011541265_1 netOGlyc-4.0.0.13 CARBOHYD 829 829 0.029793 . .
115. XP_011541265_1 netOGlyc-4.0.0.13 CARBOHYD 833 833 0.00762554 . .
116. XP_011541265_1 netOGlyc-4.0.0.13 CARBOHYD 843 843 0.00428043 . .
117. XP_011541265_1 netOGlyc-4.0.0.13 CARBOHYD 868 868 0.0116004 . .
118. XP_011541265_1 netOGlyc-4.0.0.13 CARBOHYD 872 872 0.0567303 . .
119. XP_011541265_1 netOGlyc-4.0.0.13 CARBOHYD 873 873 0.0211881 . .
120. XP_011541265_1 netOGlyc-4.0.0.13 CARBOHYD 893 893 0.0251094 . .
121. XP_011541265_1 netOGlyc-4.0.0.13 CARBOHYD 905 905 0.0658499 . .
122. XP_011541265_1 netOGlyc-4.0.0.13 CARBOHYD 910 910 0.0364509 . .
123. XP_011541265_1 netOGlyc-4.0.0.13 CARBOHYD 917 917 0.0962546 . .
124. XP_011541265_1 netOGlyc-4.0.0.13 CARBOHYD 927 927 0.0716698 . .
125. XP_011541265_1 netOGlyc-4.0.0.13 CARBOHYD 936 936 0.0466306 . .
126. XP_011541265_1 netOGlyc-4.0.0.13 CARBOHYD 947 947 0.152202 . .
127. XP_011541265_1 netOGlyc-4.0.0.13 CARBOHYD 949 949 0.0508989 . .
128. XP_011541265_1 netOGlyc-4.0.0.13 CARBOHYD 954 954 0.0759783 . .
129. XP_011541265_1 netOGlyc-4.0.0.13 CARBOHYD 979 979 0.0251436 . .
130. XP_011541265_1 netOGlyc-4.0.0.13 CARBOHYD 982 982 0.0372035 . .
131. XP_011541265_1 netOGlyc-4.0.0.13 CARBOHYD 987 987 0.285375 . .
132. XP_011541265_1 netOGlyc-4.0.0.13 CARBOHYD 990 990 0.366093 . .
133. XP_011541265_1 netOGlyc-4.0.0.13 CARBOHYD 992 992 0.0796826 . .
134. XP_011541265_1 netOGlyc-4.0.0.13 CARBOHYD 1002 1002 0.111133 . .
135. XP_011541265_1 netOGlyc-4.0.0.13 CARBOHYD 1016 1016 0.300373 . .
136. XP_011541265_1 netOGlyc-4.0.0.13 CARBOHYD 1018 1018 0.441835 . .
137. XP_011541265_1 netOGlyc-4.0.0.13 CARBOHYD 1027 1027 0.263806 . .
138. XP_011541265_1 netOGlyc-4.0.0.13 CARBOHYD 1037 1037 0.405608 . .
139. XP_011541265_1 netOGlyc-4.0.0.13 CARBOHYD 1039 1039 0.597296 . . #POSITIVE
140. XP_011541265_1 netOGlyc-4.0.0.13 CARBOHYD 1047 1047 0.839499 . . #POSITIVE
141. XP_011541265_1 netOGlyc-4.0.0.13 CARBOHYD 1048 1048 0.546308 . . #POSITIVE
142. XP_011541265_1 netOGlyc-4.0.0.13 CARBOHYD 1049 1049 0.749442 . . #POSITIVE
143. XP_011541265_1 netOGlyc-4.0.0.13 CARBOHYD 1053 1053 0.655093 . . #POSITIVE
144. XP_011541265_1 netOGlyc-4.0.0.13 CARBOHYD 1070 1070 0.666779 . . #POSITIVE
145. XP_011541265_1 netOGlyc-4.0.0.13 CARBOHYD 1076 1076 0.721474 . . #POSITIVE
146. XP_011541265_1 netOGlyc-4.0.0.13 CARBOHYD 1077 1077 0.637751 . . #POSITIVE
147. XP_011541265_1 netOGlyc-4.0.0.13 CARBOHYD 1083 1083 0.143701 . .
148. XP_011541265_1 netOGlyc-4.0.0.13 CARBOHYD 1090 1090 0.432704 . .
149. XP_011541265_1 netOGlyc-4.0.0.13 CARBOHYD 1092 1092 0.47458 . .
150. XP_011541265_1 netOGlyc-4.0.0.13 CARBOHYD 1097 1097 0.154372 . .
151. XP_011541265_1 netOGlyc-4.0.0.13 CARBOHYD 1110 1110 0.183722 . .
152. XP_011541265_1 netOGlyc-4.0.0.13 CARBOHYD 1113 1113 0.100889 . .
153. XP_011541265_1 netOGlyc-4.0.0.13 CARBOHYD 1122 1122 0.241942 . .
154. XP_011541265_1 netOGlyc-4.0.0.13 CARBOHYD 1128 1128 0.236755 . .
155. XP_011541265_1 netOGlyc-4.0.0.13 CARBOHYD 1129 1129 0.303914 . .
156. XP_011541265_1 netOGlyc-4.0.0.13 CARBOHYD 1135 1135 0.208782 . .
157. XP_011541265_1 netOGlyc-4.0.0.13 CARBOHYD 1136 1136 0.361262 . .
158. XP_011541265_1 netOGlyc-4.0.0.13 CARBOHYD 1149 1149 0.310005 . .
159. XP_011541265_1 netOGlyc-4.0.0.13 CARBOHYD 1155 1155 0.388476 . .
160. XP_011541265_1 netOGlyc-4.0.0.13 CARBOHYD 1160 1160 0.619084 . . #POSITIVE
161. XP_011541265_1 netOGlyc-4.0.0.13 CARBOHYD 1164 1164 0.600072 . . #POSITIVE
162. XP_011541265_1 netOGlyc-4.0.0.13 CARBOHYD 1184 1184 0.125836 . .
163. XP_011541265_1 netOGlyc-4.0.0.13 CARBOHYD 1190 1190 0.474985 . .
164. XP_011541265_1 netOGlyc-4.0.0.13 CARBOHYD 1210 1210 0.465567 . .
165. XP_011541265_1 netOGlyc-4.0.0.13 CARBOHYD 1214 1214 0.284921 . .
166. XP_011541265_1 netOGlyc-4.0.0.13 CARBOHYD 1215 1215 0.511508 . . #POSITIVE
167. XP_011541265_1 netOGlyc-4.0.0.13 CARBOHYD 1225 1225 0.649886 . . #POSITIVE
168. XP_011541265_1 netOGlyc-4.0.0.13 CARBOHYD 1228 1228 0.320735 . .
169. XP_011541265_1 netOGlyc-4.0.0.13 CARBOHYD 1238 1238 0.303617 . .
170. XP_011541265_1 netOGlyc-4.0.0.13 CARBOHYD 1255 1255 0.199175 . .
171. XP_011541265_1 netOGlyc-4.0.0.13 CARBOHYD 1268 1268 0.332453 . .
172. XP_011541265_1 netOGlyc-4.0.0.13 CARBOHYD 1278 1278 0.720646 . . #POSITIVE
173. XP_011541265_1 netOGlyc-4.0.0.13 CARBOHYD 1301 1301 0.174956 . .
174. XP_011541265_1 netOGlyc-4.0.0.13 CARBOHYD 1316 1316 0.143501 . .
175. XP_011541265_1 netOGlyc-4.0.0.13 CARBOHYD 1325 1325 0.631733 . . #POSITIVE
176. XP_011541265_1 netOGlyc-4.0.0.13 CARBOHYD 1331 1331 0.521625 . . #POSITIVE
177. XP_011541265_1 netOGlyc-4.0.0.13 CARBOHYD 1360 1360 0.144186 . .
178. XP_011541265_1 netOGlyc-4.0.0.13 CARBOHYD 1368 1368 0.452739 . .
179. XP_011541265_1 netOGlyc-4.0.0.13 CARBOHYD 1374 1374 0.91782 . . #POSITIVE
180. XP_011541265_1 netOGlyc-4.0.0.13 CARBOHYD 1375 1375 0.941535 . . #POSITIVE
181. XP_011541265_1 netOGlyc-4.0.0.13 CARBOHYD 1379 1379 0.761992 . . #POSITIVE
182. XP_011541265_1 netOGlyc-4.0.0.13 CARBOHYD 1380 1380 0.650536 . . #POSITIVE
183. XP_011541265_1 netOGlyc-4.0.0.13 CARBOHYD 1389 1389 0.445755 . .
184. XP_011541265_1 netOGlyc-4.0.0.13 CARBOHYD 1390 1390 0.575199 . . #POSITIVE
185. XP_011541265_1 netOGlyc-4.0.0.13 CARBOHYD 1392 1392 0.244409 . .
186. XP_011541265_1 netOGlyc-4.0.0.13 CARBOHYD 1397 1397 0.365735 . .
187. XP_011541265_1 netOGlyc-4.0.0.13 CARBOHYD 1410 1410 0.123068 . .
188. XP_011541265_1 netOGlyc-4.0.0.13 CARBOHYD 1422 1422 0.9224 . . #POSITIVE
189. XP_011541265_1 netOGlyc-4.0.0.13 CARBOHYD 1425 1425 0.96346 . . #POSITIVE
190. XP_011541265_1 netOGlyc-4.0.0.13 CARBOHYD 1426 1426 0.913108 . . #POSITIVE
191. XP_011541265_1 netOGlyc-4.0.0.13 CARBOHYD 1428 1428 0.804818 . . #POSITIVE
192. XP_011541265_1 netOGlyc-4.0.0.13 CARBOHYD 1445 1445 0.247152 . .
193. XP_011541265_1 netOGlyc-4.0.0.13 CARBOHYD 1470 1470 0.87903 . . #POSITIVE
194. XP_011541265_1 netOGlyc-4.0.0.13 CARBOHYD 1472 1472 0.776309 . . #POSITIVE
195. XP_011541265_1 netOGlyc-4.0.0.13 CARBOHYD 1473 1473 0.651327 . . #POSITIVE
196. XP_011541265_1 netOGlyc-4.0.0.13 CARBOHYD 1475 1475 0.424694 . .
197. XP_011541265_1 netOGlyc-4.0.0.13 CARBOHYD 1478 1478 0.525682 . . #POSITIVE
198. XP_011541265_1 netOGlyc-4.0.0.13 CARBOHYD 1493 1493 0.0552486 . .
199. XP_011541265_1 netOGlyc-4.0.0.13 CARBOHYD 1503 1503 0.228007 . .
200. XP_011541265_1 netOGlyc-4.0.0.13 CARBOHYD 1506 1506 0.098691 . .
201. XP_011541265_1 netOGlyc-4.0.0.13 CARBOHYD 1512 1512 0.50607 . . #POSITIVE
202. XP_011541265_1 netOGlyc-4.0.0.13 CARBOHYD 1516 1516 0.18434 . .
203. XP_011541265_1 netOGlyc-4.0.0.13 CARBOHYD 1526 1526 0.614639 . . #POSITIVE
204. XP_011541265_1 netOGlyc-4.0.0.13 CARBOHYD 1530 1530 0.389352 . .
205. XP_011541265_1 netOGlyc-4.0.0.13 CARBOHYD 1534 1534 0.52332 . . #POSITIVE
206. XP_011541265_1 netOGlyc-4.0.0.13 CARBOHYD 1536 1536 0.717469 . . #POSITIVE
207. XP_011541265_1 netOGlyc-4.0.0.13 CARBOHYD 1545 1545 0.860578 . . #POSITIVE
208. XP_011541265_1 netOGlyc-4.0.0.13 CARBOHYD 1547 1547 0.718211 . . #POSITIVE
209. XP_011541265_1 netOGlyc-4.0.0.13 CARBOHYD 1560 1560 0.466345 . .
210. XP_011541265_1 netOGlyc-4.0.0.13 CARBOHYD 1564 1564 0.706507 . . #POSITIVE
211. XP_011541265_1 netOGlyc-4.0.0.13 CARBOHYD 1567 1567 0.422722 . .
212. XP_011541265_1 netOGlyc-4.0.0.13 CARBOHYD 1569 1569 0.433645 . .
213. XP_011541265_1 netOGlyc-4.0.0.13 CARBOHYD 1572 1572 0.270858 . .
214. XP_011541265_1 netOGlyc-4.0.0.13 CARBOHYD 1574 1574 0.317131 . .
215. XP_011541265_1 netOGlyc-4.0.0.13 CARBOHYD 1583 1583 0.143111 . .
216. XP_011541265_1 netOGlyc-4.0.0.13 CARBOHYD 1584 1584 0.21566 . .
217. XP_011541265_1 netOGlyc-4.0.0.13 CARBOHYD 1595 1595 0.0751513 . .
218. XP_011541265_1 netOGlyc-4.0.0.13 CARBOHYD 1600 1600 0.257083 . .
219. XP_011541265_1 netOGlyc-4.0.0.13 CARBOHYD 1605 1605 0.488393 . .
220. XP_011541265_1 netOGlyc-4.0.0.13 CARBOHYD 1608 1608 0.225697 . .
221. XP_011541265_1 netOGlyc-4.0.0.13 CARBOHYD 1622 1622 0.154678 . .
222. XP_011541265_1 netOGlyc-4.0.0.13 CARBOHYD 1641 1641 0.0581102 . .
223. XP_011541265_1 netOGlyc-4.0.0.13 CARBOHYD 1653 1653 0.0817604 . .
224. XP_011541265_1 netOGlyc-4.0.0.13 CARBOHYD 1655 1655 0.0433279 . .
225. XP_011541265_1 netOGlyc-4.0.0.13 CARBOHYD 1657 1657 0.0091501 . .
226. XP_011541265_1 netOGlyc-4.0.0.13 CARBOHYD 1662 1662 0.317465 . .
227. XP_011541265_1 netOGlyc-4.0.0.13 CARBOHYD 1667 1667 0.127056 . .
228. XP_011541265_1 netOGlyc-4.0.0.13 CARBOHYD 1670 1670 0.0293271 . .
229. XP_011541265_1 netOGlyc-4.0.0.13 CARBOHYD 1679 1679 0.0598197 . .
230. XP_011541265_1 netOGlyc-4.0.0.13 CARBOHYD 1682 1682 0.0855592 . .
231. XP_011541265_1 netOGlyc-4.0.0.13 CARBOHYD 1689 1689 0.317299 . .
232. XP_011541265_1 netOGlyc-4.0.0.13 CARBOHYD 1690 1690 0.0831073 . .
233. XP_011541265_1 netOGlyc-4.0.0.13 CARBOHYD 1697 1697 0.401599 . .
234. XP_011541265_1 netOGlyc-4.0.0.13 CARBOHYD 1699 1699 0.361589 . .
235. XP_011541265_1 netOGlyc-4.0.0.13 CARBOHYD 1701 1701 0.179246 . .
236. XP_011541265_1 netOGlyc-4.0.0.13 CARBOHYD 1703 1703 0.206521 . .
237. XP_011541265_1 netOGlyc-4.0.0.13 CARBOHYD 1704 1704 0.295793 . .
238. XP_011541265_1 netOGlyc-4.0.0.13 CARBOHYD 1719 1719 0.0534059 . .
239. XP_011541265_1 netOGlyc-4.0.0.13 CARBOHYD 1726 1726 0.0469076 . .
240. XP_011541265_1 netOGlyc-4.0.0.13 CARBOHYD 1728 1728 0.0173377 . .
241. XP_011541265_1 netOGlyc-4.0.0.13 CARBOHYD 1730 1730 0.00557004 . .
242. XP_011541265_1 netOGlyc-4.0.0.13 CARBOHYD 1733 1733 0.00944661 . .
243. XP_011541265_1 netOGlyc-4.0.0.13 CARBOHYD 1750 1750 0.132648 . .
244. XP_011541265_1 netOGlyc-4.0.0.13 CARBOHYD 1757 1757 0.348542 . .
245. XP_011541265_1 netOGlyc-4.0.0.13 CARBOHYD 1763 1763 0.0661772 . .
246. XP_011541265_1 netOGlyc-4.0.0.13 CARBOHYD 1766 1766 0.201487 . .
247. XP_011541265_1 netOGlyc-4.0.0.13 CARBOHYD 1773 1773 0.0427351 . .
248. XP_011541265_1 netOGlyc-4.0.0.13 CARBOHYD 1776 1776 0.037824 . .
249. XP_011541265_1 netOGlyc-4.0.0.13 CARBOHYD 1777 1777 0.187887 . .
250. XP_011541265_1 netOGlyc-4.0.0.13 CARBOHYD 1781 1781 0.109389 . .
251. XP_011541265_1 netOGlyc-4.0.0.13 CARBOHYD 1786 1786 0.0252465 . .
252. XP_011541265_1 netOGlyc-4.0.0.13 CARBOHYD 1791 1791 0.282632 . .
253. XP_011541265_1 netOGlyc-4.0.0.13 CARBOHYD 1800 1800 0.368713 . .
254. XP_011541265_1 netOGlyc-4.0.0.13 CARBOHYD 1809 1809 0.583246 . . #POSITIVE
255. XP_011541265_1 netOGlyc-4.0.0.13 CARBOHYD 1818 1818 0.0947202 . .
256. XP_011541265_1 netOGlyc-4.0.0.13 CARBOHYD 1823 1823 0.0417919 . .
257. XP_011541265_1 netOGlyc-4.0.0.13 CARBOHYD 1825 1825 0.0176966 . .
258. XP_011541265_1 netOGlyc-4.0.0.13 CARBOHYD 1838 1838 0.13006 . .
259. XP_011541265_1 netOGlyc-4.0.0.13 CARBOHYD 1839 1839 0.112004 . .
260. XP_011541265_1 netOGlyc-4.0.0.13 CARBOHYD 1849 1849 0.34967 . .
261. XP_011541265_1 netOGlyc-4.0.0.13 CARBOHYD 1851 1851 0.188122 . .
262. XP_011541265_1 netOGlyc-4.0.0.13 CARBOHYD 1852 1852 0.0534808 . .
263. XP_011541265_1 netOGlyc-4.0.0.13 CARBOHYD 1853 1853 0.112344 . .
264. XP_011541265_1 netOGlyc-4.0.0.13 CARBOHYD 1858 1858 0.0908196 . .
265. XP_011541265_1 netOGlyc-4.0.0.13 CARBOHYD 1862 1862 0.08196 . .
266. XP_011541265_1 netOGlyc-4.0.0.13 CARBOHYD 1881 1881 0.0773247 . .
267. XP_011541265_1 netOGlyc-4.0.0.13 CARBOHYD 1882 1882 0.162604 . .
268. XP_011541265_1 netOGlyc-4.0.0.13 CARBOHYD 1893 1893 0.562159 . . #POSITIVE
269. XP_011541265_1 netOGlyc-4.0.0.13 CARBOHYD 1905 1905 0.408197 . .
270. XP_011541265_1 netOGlyc-4.0.0.13 CARBOHYD 1908 1908 0.242705 . .
271. XP_011541265_1 netOGlyc-4.0.0.13 CARBOHYD 1909 1909 0.351798 . .
272. XP_011541265_1 netOGlyc-4.0.0.13 CARBOHYD 1916 1916 0.0644142 . .
273. XP_011541265_1 netOGlyc-4.0.0.13 CARBOHYD 1920 1920 0.0284854 . .
274. XP_011541265_1 netOGlyc-4.0.0.13 CARBOHYD 1938 1938 0.109255 . .
275. XP_011541265_1 netOGlyc-4.0.0.13 CARBOHYD 1941 1941 0.160911 . .
276. XP_011541265_1 netOGlyc-4.0.0.13 CARBOHYD 1946 1946 0.522298 . . #POSITIVE
277. XP_011541265_1 netOGlyc-4.0.0.13 CARBOHYD 1949 1949 0.275535 . .
278. XP_011541265_1 netOGlyc-4.0.0.13 CARBOHYD 1950 1950 0.19353 . .
279. XP_011541265_1 netOGlyc-4.0.0.13 CARBOHYD 1954 1954 0.0750492 . .
280. XP_011541265_1 netOGlyc-4.0.0.13 CARBOHYD 1974 1974 0.0748254 . .
281. XP_011541265_1 netOGlyc-4.0.0.13 CARBOHYD 1977 1977 0.143987 . .
282. XP_011541265_1 netOGlyc-4.0.0.13 CARBOHYD 1978 1978 0.155633 . .
283. XP_011541265_1 netOGlyc-4.0.0.13 CARBOHYD 1982 1982 0.299501 . .
284. XP_011541265_1 netOGlyc-4.0.0.13 CARBOHYD 1983 1983 0.373764 . .
285. XP_011541265_1 netOGlyc-4.0.0.13 CARBOHYD 1985 1985 0.697176 . . #POSITIVE
286. XP_011541265_1 netOGlyc-4.0.0.13 CARBOHYD 1987 1987 0.301533 . .
287. XP_011541265_1 netOGlyc-4.0.0.13 CARBOHYD 1996 1996 0.131349 . .
288. XP_011541265_1 netOGlyc-4.0.0.13 CARBOHYD 2007 2007 0.0605304 . .
289. XP_011541265_1 netOGlyc-4.0.0.13 CARBOHYD 2018 2018 0.0654117 . .
290. XP_011541265_1 netOGlyc-4.0.0.13 CARBOHYD 2028 2028 0.0315355 . .
291. XP_011541265_1 netOGlyc-4.0.0.13 CARBOHYD 2033 2033 0.0266311 . .
292. XP_011541265_1 netOGlyc-4.0.0.13 CARBOHYD 2039 2039 0.0828575 . .
293. XP_011541265_1 netOGlyc-4.0.0.13 CARBOHYD 2040 2040 0.0223196 . .
294. XP_011541265_1 netOGlyc-4.0.0.13 CARBOHYD 2047 2047 0.0361072 . .
295. XP_011541265_1 netOGlyc-4.0.0.13 CARBOHYD 2056 2056 0.0176091 . .
296. XP_011541265_1 netOGlyc-4.0.0.13 CARBOHYD 2058 2058 0.0227079 . .
297. XP_011541265_1 netOGlyc-4.0.0.13 CARBOHYD 2083 2083 0.481513 . .
298. XP_011541265_1 netOGlyc-4.0.0.13 CARBOHYD 2088 2088 0.220177 . .
299. XP_011541265_1 netOGlyc-4.0.0.13 CARBOHYD 2090 2090 0.274294 . .
300. XP_011541265_1 netOGlyc-4.0.0.13 CARBOHYD 2095 2095 0.277345 . .
301. XP_011541265_1 netOGlyc-4.0.0.13 CARBOHYD 2096 2096 0.232837 . .
302. XP_011541265_1 netOGlyc-4.0.0.13 CARBOHYD 2112 2112 0.00392419 . .
303. XP_011541265_1 netOGlyc-4.0.0.13 CARBOHYD 2122 2122 0.00551842 . .
304. XP_011541265_1 netOGlyc-4.0.0.13 CARBOHYD 2129 2129 0.0132868 . .
305. XP_011541265_1 netOGlyc-4.0.0.13 CARBOHYD 2130 2130 0.0160756 . .
306. XP_011541265_1 netOGlyc-4.0.0.13 CARBOHYD 2132 2132 0.00946387 . .
307. XP_011541265_1 netOGlyc-4.0.0.13 CARBOHYD 2137 2137 0.014119 . .
308. XP_011541265_1 netOGlyc-4.0.0.13 CARBOHYD 2140 2140 0.00344423 . .
309. XP_011541265_1 netOGlyc-4.0.0.13 CARBOHYD 2141 2141 0.00853672 . .
310. XP_011541265_1 netOGlyc-4.0.0.13 CARBOHYD 2145 2145 0.00424052 . .
311. XP_011541265_1 netOGlyc-4.0.0.13 CARBOHYD 2149 2149 0.00516429 . .
312. XP_011541265_1 netOGlyc-4.0.0.13 CARBOHYD 2150 2150 0.0184868 . .
313. XP_011541265_1 netOGlyc-4.0.0.13 CARBOHYD 2165 2165 0.0132893 . .
314. XP_011541265_1 netOGlyc-4.0.0.13 CARBOHYD 2168 2168 0.0457966 . .

**>NP_009053.1 upstream stimulatory factor 1 isoform 1 (USF1) [Homo sapiens]**

MKGQQKTAETEEGTVQIQEGAVATGEDPTSVAIASIQSAATFPDPNVKYVFRTENGGQVMYRVIQVSEGQ

LDGQTEGTGAISGYPATQSMTQAVIQGAFTSDDAVDTEGTAAETHYTYFPSTAVGDGAGGTTSGSTAAVV

TTQGSEALLGQATPPGTGQFFVMMSPQEVLQGGSQRSIAPRTHPYSPKSEAPRTTRDEKRRAQHNEVERR

RRDKINNWIVQLSKIIPDCSMESTKSGQSKGGILSKACDYIQELRQSNHRLSEELQGLDQLQLDNDVLRQ

QVEDLKNKNLLLRAQLRHHGLEVVIKNDSN

**USF1 S-nitrosylation Data**

Postion Peptide Score cutoff cluster

229 LSKIIPDCSMESTKS 17.781 0 Cluster C

248 GGILSKACDYIQELR 1.284 0 Cluster A

**USF1 Phosphorylation DATA**

>NP_009053.1 310 amino acids

**# netphos-3.1b prediction results**

# Sequence # x Context Score Kinase Answer

-------------------------------------------------------------------

1. # NP_009053.1 10 T KTAETEEGT 0.823 unsp YES
2. # NP_009053.1 24 T GAVATGEDP 0.520 unsp YES
3. # NP_009053.1 41 T QSAATFPDP 0.555 PKC YES
4. # NP_009053.1 49 Y PNVKYVFRT 0.868 unsp YES
5. # NP_009053.1 67 S VIQVSEGQL 0.928 unsp YES
6. # NP_009053.1 82 S TGAISGYPA 0.791 unsp YES
7. # NP_009053.1 82 S TGAISGYPA 0.558 cdc2 YES
8. # NP_009053.1 87 T GYPATQSMT 0.583 DNAPK YES
9. # NP_009053.1 89 S PATQSMTQA 0.570 PKC YES
10. # NP_009053.1 91 T TQSMTQAVI 0.625 DNAPK YES
11. # NP_009053.1 91 T TQSMTQAVI 0.503 ATM YES
12. # NP_009053.1 100 T QGAFTSDDA 0.858 unsp YES
13. # NP_009053.1 100 T QGAFTSDDA 0.549 CKII YES
14. # NP_009053.1 101 S GAFTSDDAV 0.552 CKII YES
15. # NP_009053.1 107 T DAVDTEGTA 0.769 unsp YES
16. # NP_009053.1 107 T DAVDTEGTA 0.608 CKII YES
17. # NP_009053.1 107 T DAVDTEGTA 0.538 CKI YES
18. # NP_009053.1 110 T DTEGTAAET 0.962 unsp YES
19. # NP_009053.1 110 T DTEGTAAET 0.520 CKII YES
20. # NP_009053.1 116 Y AETHYTYFP 0.957 unsp YES
21. # NP_009053.1 122 T YFPSTAVGD 0.597 unsp YES
22. # NP_009053.1 131 T GAGGTTSGS 0.719 PKC YES
23. # NP_009053.1 132 T AGGTTSGST 0.735 unsp YES
24. # NP_009053.1 153 T LGQATPPGT 0.767 unsp YES
25. # NP_009053.1 153 T LGQATPPGT 0.507 p38MAPK YES
26. # NP_009053.1 165 S FVMMSPQEV 0.555 p38MAPK YES
27. # NP_009053.1 174 S LQGGSQRSI 0.567 DNAPK YES
28. # NP_009053.1 182 T IAPRTHPYS 0.657 PKC YES
29. # NP_009053.1 186 S THPYSPKSE 0.997 unsp YES
30. # NP_009053.1 186 S THPYSPKSE 0.620 cdk5 YES
31. # NP_009053.1 186 S THPYSPKSE 0.517 GSK3 YES
32. # NP_009053.1 189 S YSPKSEAPR 0.668 unsp YES
33. # NP_009053.1 194 T EAPRTTRDE 0.974 unsp YES
34. # NP_009053.1 194 T EAPRTTRDE 0.683 PKC YES
35. # NP_009053.1 195 T APRTTRDEK 0.984 unsp YES
36. # NP_009053.1 195 T APRTTRDEK 0.514 PKG YES
37. # NP_009053.1 195 T APRTTRDEK 0.500 CKII YES
38.
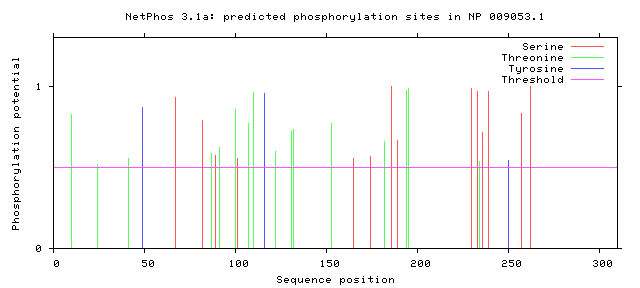
# NP_009053.1 230 S IPDCSMEST 0.983 unsp YES
39. # NP_009053.1 233 S CSMESTKSG 0.969 unsp YES
40. # NP_009053.1 233 S CSMESTKSG 0.734 PKC YES
41. # NP_009053.1 234 T SMESTKSGQ 0.535 CKI YES
42. # NP_009053.1 236 S ESTKSGQSK 0.712 PKC YES
43. # NP_009053.1 239 S KSGQSKGGI 0.966 unsp YES
44. # NP_009053.1 239 S KSGQSKGGI 0.587 PKC YES
45. # NP_009053.1 239 S KSGQSKGGI 0.526 CKI YES
46. # NP_009053.1 250 Y KACDYIQEL 0.543 EGFR YES
47. # NP_009053.1 257 S ELRQSNHRL 0.833 unsp YES
48. # NP_009053.1 257 S ELRQSNHRL 0.563 PKA YES
49. # NP_009053.1 262 S NHRLSEELQ 0.996 unsp YES

**USF1 Glycosylation DATA**

**NetOGlyc**

##gff-version 2

##source-version NetOGlyc 4.0.0.13

##date 18-9-7

##Type Protein

#seqname source feature start end score strand frame comment

1. NP_009053_1 netOGlyc-4.0.0.13 CARBOHYD 7 7 0.861576 . . #POSITIVE
2. NP_009053_1 netOGlyc-4.0.0.13 CARBOHYD 10 10 0.91674 . . #POSITIVE
3. NP_009053_1 netOGlyc-4.0.0.13 CARBOHYD 14 14 0.77079 . . #POSITIVE
4. NP_009053_1 netOGlyc-4.0.0.13 CARBOHYD 24 24 0.853304 . . #POSITIVE
5. NP_009053_1 netOGlyc-4.0.0.13 CARBOHYD 29 29 0.636348 . . #POSITIVE
6. NP_009053_1 netOGlyc-4.0.0.13 CARBOHYD 30 30 0.825692 . . #POSITIVE
7. NP_009053_1 netOGlyc-4.0.0.13 CARBOHYD 35 35 0.71038 . . #POSITIVE
8. NP_009053_1 netOGlyc-4.0.0.13 CARBOHYD 38 38 0.831725 . . #POSITIVE
9. NP_009053_1 netOGlyc-4.0.0.13 CARBOHYD 41 41 0.824603 . . #POSITIVE
10. NP_009053_1 netOGlyc-4.0.0.13 CARBOHYD 53 53 0.544932 . . #POSITIVE
11. NP_009053_1 netOGlyc-4.0.0.13 CARBOHYD 67 67 0.654829 . . #POSITIVE
12. NP_009053_1 netOGlyc-4.0.0.13 CARBOHYD 75 75 0.681089 . . #POSITIVE
13. NP_009053_1 netOGlyc-4.0.0.13 CARBOHYD 78 78 0.557763 . . #POSITIVE
14. NP_009053_1 netOGlyc-4.0.0.13 CARBOHYD 82 82 0.887978 . . #POSITIVE
15. NP_009053_1 netOGlyc-4.0.0.13 CARBOHYD 87 87 0.5 . . #POSITIVE
16. NP_009053_1 netOGlyc-4.0.0.13 CARBOHYD 89 89 0.623981 . . #POSITIVE
17. NP_009053_1 netOGlyc-4.0.0.13 CARBOHYD 91 91 0.645988 . . #POSITIVE
18. NP_009053_1 netOGlyc-4.0.0.13 CARBOHYD 100 100 0.866377 . . #POSITIVE
19. NP_009053_1 netOGlyc-4.0.0.13 CARBOHYD 101 101 0.850811 . . #POSITIVE
20. NP_009053_1 netOGlyc-4.0.0.13 CARBOHYD 107 107 0.877347 . . #POSITIVE
21. NP_009053_1 netOGlyc-4.0.0.13 CARBOHYD 110 110 0.874183 . . #POSITIVE
22. NP_009053_1 netOGlyc-4.0.0.13 CARBOHYD 114 114 0.939192 . . #POSITIVE
23. NP_009053_1 netOGlyc-4.0.0.13 CARBOHYD 117 117 0.875665 . . #POSITIVE
24. NP_009053_1 netOGlyc-4.0.0.13 CARBOHYD 121 121 0.973429 . . #POSITIVE
25. NP_009053_1 netOGlyc-4.0.0.13 CARBOHYD 122 122 0.905516 . . #POSITIVE
26. NP_009053_1 netOGlyc-4.0.0.13 CARBOHYD 131 131 0.932212 . . #POSITIVE
27. NP_009053_1 netOGlyc-4.0.0.13 CARBOHYD 132 132 0.968873 . . #POSITIVE
28. NP_009053_1 netOGlyc-4.0.0.13 CARBOHYD 133 133 0.955633 . . #POSITIVE
29. NP_009053_1 netOGlyc-4.0.0.13 CARBOHYD 135 135 0.980023 . . #POSITIVE
30. NP_009053_1 netOGlyc-4.0.0.13 CARBOHYD 136 136 0.973456 . . #POSITIVE
31. NP_009053_1 netOGlyc-4.0.0.13 CARBOHYD 141 141 0.957878 . . #POSITIVE
32. NP_009053_1 netOGlyc-4.0.0.13 CARBOHYD 142 142 0.950293 . . #POSITIVE
33. NP_009053_1 netOGlyc-4.0.0.13 CARBOHYD 145 145 0.921202 . . #POSITIVE
34. NP_009053_1 netOGlyc-4.0.0.13 CARBOHYD 153 153 0.912145 . . #POSITIVE
35. NP_009053_1 netOGlyc-4.0.0.13 CARBOHYD 157 157 0.862348 . . #POSITIVE
36. NP_009053_1 netOGlyc-4.0.0.13 CARBOHYD 165 165 0.791657 . . #POSITIVE
37. NP_009053_1 netOGlyc-4.0.0.13 CARBOHYD 174 174 0.942414 . . #POSITIVE
38. NP_009053_1 netOGlyc-4.0.0.13 CARBOHYD 177 177 0.973197 . . #POSITIVE
39. NP_009053_1 netOGlyc-4.0.0.13 CARBOHYD 182 182 0.924276 . . #POSITIVE
40. NP_009053_1 netOGlyc-4.0.0.13 CARBOHYD 186 186 0.993252 . . #POSITIVE
41. NP_009053_1 netOGlyc-4.0.0.13 CARBOHYD 189 189 0.983362 . . #POSITIVE
42. NP_009053_1 netOGlyc-4.0.0.13 CARBOHYD 194 194 0.871611 . . #POSITIVE
43. NP_009053_1 netOGlyc-4.0.0.13 CARBOHYD 195 195 0.905789 . . #POSITIVE

**NetNGlyc**

No sites predicted in this sequence.


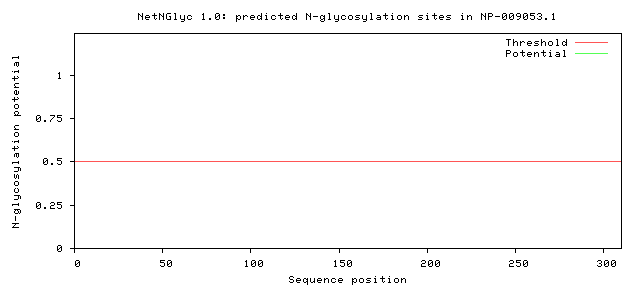

Supplement: Supplementary file 1 [file Table1.DOCX]
